# On traveling wave solutions with stability and phase plane analysis for the modified Benjamin-Bona-Mahony equation

**DOI:** 10.1371/journal.pone.0306196

**Published:** 2024-07-02

**Authors:** Md. Sagib, Md. Aslam Hossain, Bijan Krishna Saha, Kamruzzaman Khan

**Affiliations:** 1 Department of Mathematics, Hajee Mohammad Danesh Science and Technology University, Dinajpur, Bangladesh; 2 Department of Mathematics, Pabna University of Science and Technology, Pabna, Bangladesh; 3 Department of Mathematics, University of Barishal, Barishal, Bangladesh; 4 School of Science and Technology, University of New England, Armidale, NSW, Australia; Tel Aviv University, ISRAEL

## Abstract

The modified Benjamin-Bona-Mahony (mBBM) model is utilized in the optical illusion field to describe the propagation of long waves in a nonlinear dispersive medium during a visual illusion (Khater 2021). This article investigates the mBBM equation through the utilization of the rational $\left(\frac{G^{\prime }}{G}\right)$-expansion technique to derive new analytical wave solutions. The analytical solutions we have obtained comprise hyperbolic, trigonometric, and rational functions. Some of these exact solutions closely align with previously published results in specific cases, affirming the validity of our other solutions. To provide insights into diverse wave propagation characteristics, we have conducted an in-depth analysis of these solutions using 2D, 3D, and density plots. We also investigated the effects of various parameters on the characteristics of the obtained wave solutions of the model. Moreover, we employed the techniques of linear stability to perform stability analysis of the considered model. Additionally, we have explored the stability of the associated dynamical system through the application of phase plane theory. This study also demonstrates the efficacy and capabilities of the rational $\left(\frac{G^{\prime }}{G}\right)$-expansion approach in analyzing and extracting soliton solutions from nonlinear partial differential equations.

## 1. Introduction

The study of nonlinear partial differential equations (NLPDEs) is now regarded as one of the most important and fundamental areas of science, particularly for uncovering previously unknown characteristics of complex phenomena in a variety of disciplines, including chemical physics, biology, plasma physics, hydrodynamics, condensed matter physics, fluid mechanics, ocean physics, nuclear physics, optical physics, and more. In order to reflect many processes in nature, physics, and engineering, numerous nonlinear models have been developed. Numerous researchers have developed different methods and approaches to examine analytical solutions considering the significance of these models and their varied applications. The F-expansion approach [1], Lie symmetry technique [2], Kudryashov process [3], $\left(\frac{G^{\prime }}{G},\frac{1}{G}\right)$-expansion technique [4], rational sine–cosine scheme [5], modified Jacobi elliptic expansion method [6], exponential-expansion scheme [7], generalized exponential method [8], the hyperbolic function method [9], positive quadratic function method [10], the inverse scattering approach [11], generalized Khater technique [12], test function method [13], improved tanh method [14], different form of the $\left(\frac{G^{\prime }}{G}\right)$-expansion methods [15–18], and other such methods are examples of these approaches.

The study on solutions, structures, interactions, and further properties of solitary wave and soliton attained much concentration, and different meaningful results have been successfully derived [19–26]. In recent years, significant advancements have been made in the study of soliton solutions through the utilization of the Hirota bilinear method [27]. Noteworthy contributions have emerged from several recent studies, shedding new light on the method’s applicability. For example, the analysis of multi-soliton solutions and soliton molecules in the context of the (2+1)-dimensional Boiti-Leon-Manna-Pempinelli equation underscores the usefulness of the Hirota bilinear method [28]. Furthermore, the exploration of novel soliton solutions to the extended (3+1)-dimensional Jimbo-Miwa equation using the Hirota bilinear method demonstrated its effectiveness in uncovering complex nonlinear dynamics [29].

The regularized long-wave equation, commonly referred to as the Benjamin–Bona–Mahony (BBM) equation, is given by

$$\begin{array}{c} u_{t}+u_{x}+uu_{x}-u_{xxt}=0. \end{array}$$ (1)

It was originally explored by Benjamin et al. [30] as a novel model for describing long waves, providing an alternative to the Korteweg–de Vries (KdV) equation. To discover new properties of soliton waves, Eq (1) is derived as an improved model of the well-known KdV model. Significant importance is captured by this equation in the modeling of nonlinear dispersive systems. The BBM equation is applied to study hydromagnetic waves in cold plasma, long-wavelength surface waves in liquids, acoustic waves in anharmonic crystals, and acoustic-gravity waves in compressible fluids [31]. This model is utilized to study the dynamical properties of extended surface gravity waves with small amplitudes as they propagate in (1+1)-dimensions. Additionally, the BBM equation has also been used in the study of undular bores. The generalized Benjamin–Bona–Mahony equation is given by

$$\begin{array}{c} u_{t}+u_{x}+\beta u^{n}u_{x}-\gamma u_{xxt}=0,n\geq 1, \end{array}$$ (2)

where *β* is an arbitrary constant. For *β* = 1, the Eq (2) is called modified Benjamin–Bona–Mahony (mBBM) equation. In this paper, we have investigated the mBBM equation, which can be expressed as [32, 33]:

$$\begin{array}{c} u_{t}+\alpha u_{x}+\beta u^{2}u_{x}-\gamma u_{xxt}=0, \end{array}$$ (3)

where *α*, *β* and *γ* denote any real constants. Similarly, the Eq (3) can be viewed as an alternative formulation of the modified Korteweg-de Vries equation. The mBBM equation also finds application in the realm of optical illusions, where light, color, and patterns are skillfully combined to craft images capable of deceiving the human brain, as demonstrated in prior studies [34–36]. An optical illusion is defined as an illusion created by the visual system and characterized by a perception of the visual world that may appear to deviate from reality.

So far, numerous scholars have made significant contributions to solve the mBBM equation. The $\left(\frac{G^{\prime }}{G}\right)$-function method is utilized by Aslan [32] to generate exact and explicit solutions. Abbasbandy and Shirzadi [31] employed the first integral method to solve mBBM model, and the exp(−*ϕ*(*ξ*))-expansion process was employed by Khan and Akbar [37] for its study. Some new solitonary solutions for the mBBM model were obtained by Noor et al. [38] using exp-function method. Song [39] employed the bifurcation method of dynamical system to investigate the problem, while Tariq and Seadawy [40] adopted the auxiliary equation method to ascertain exact solutions. In two different studies, Wang [41, 42] investigated the mBBM model using different methodologies to obtain various wave structures, including bright waves, bright-dark waves, bright-like waves, kinky-bright waves, and periodic waves. Furthermore, Khater et al. [34] utilized the modified Khater method, and Naeem et al. [43] introduced the Sardar-subequation method to uncover abundant exact solutions for mBBM equation. Additionally, Khater and Salama [44] applied the modified Khater method to determine its solutions. To date, the mBBM Eq (1) has not been explored by the rational $\left(\frac{G^{\prime }}{G}\right)$-expansion method [16].

The goal of this research is to identify new analytical wave solutions, such as kink, anti-kink, anti-peakon, singular periodic, anti-bell and V-shaped soliton solutions of the mBBM model (3) through the rational $\left(\frac{G^{\prime }}{G}\right)$-expansion method. Varying parameters can cause changes in the qualitative structure of the flow. This can result in the creation or destruction of fixed points, or changes in their stability. These changes in dynamics are known as bifurcations, and the specific parameter values at which they occur are called bifurcation points. Bifurcations are significant as they offer a model for understanding transitions and instabilities that occur when a control parameter is changed. Studying the movement of orbits in a phase plane can offer valuable insights into the dynamics of the system and aid in forecasting its future behavior. By examining both the behavior of the orbit in the phase plane and the corresponding solutions of the components with respect to the wave variable, one can gain a comprehensive understanding of the system’s dynamics and make predictions about its future behavior. Furthermore, we thoroughly investigate the stability of the model, analyze the parametric effects on the wave solutions, and conduct a comprehensive phase-plane analysis to gain profound insights into complex systems across diverse scientific domains.

This paper is constructed into the subsequent sections. We have introduced the rational $\left(\frac{G^{\prime }}{G}\right)$-expansion method in Section 2 to find exact solitary wave solutions from NLPDE. In Section 3, we applied this method to solve the mBBM equation to obtain soliton solutions. Section 4 offers a graphical interpretation of the uncovered solutions to discuss their characteristics. Section 5 delves into an examination of the stability of the mBBM equation and compares our solutions with those obtained using other methods. Section 6 demonstrates the effects of different parameters on wave profiles. Finally, before concluding the discussion, Section 7 explores phase plane analysis.

## 2. Brief outline of the rational $\left(\frac{G^{\prime }}{G}\right)$-expansion method

Consider the NLPDE featuring independent variables *x*, *y*, *z*, ⋯, *t* and the dependent variable *u* as

$$\begin{array}{c} P(u,u_{t},u_{x},u_{y},u_{z},\cdots ,u_{xt},u_{yt},u_{zt},\cdots ,u_{tt},u_{xx},u_{yy},u_{zz},\cdots )=0, \end{array}$$ (4)

where *P* represents a polynomial in its variables, encompassing the highest-order nonlinear and derivative terms. The transformation *u*(*x*, *t*) = *V*(*ζ*), *ζ* = *lx* + *ωt* converts Eq (4) to the ordinary differential equation (ODE)

$$\begin{array}{c} Q(V,V^{\prime },V^{\prime \prime },V^{\prime \prime \prime },\cdots )=0, \end{array}$$ (5)

where prime denotes the differentiation with respect to *ζ*. According to the rational $\left(\frac{G^{\prime }}{G}\right)$-expansion method, the solution of Eq (3) is assumed as follows:

$$\begin{array}{c} V\left(\zeta \right)=\frac{\sum_{j=0}^{n}\alpha_{j}{\left(\frac{G^{\prime }}{G}\right)}^{n}}{\sum_{j=0}^{n}\beta_{j}{\left(\frac{G^{\prime }}{G}\right)}^{n}}, \end{array}$$ (6)

where *α*_*j*_, *β*_*j*_ (*j* = 1, 2, ⋯, *n*) are all unknown constants and *G* = *G*(*ζ*) is the solution of the ensuing ODE:

$$\begin{array}{c} G^{\prime \prime }+\nu G^{\prime }+\mu G=0, \end{array}$$ (7)

where *ν* and *μ* are any constants. The general solution of Eq (7) is

$$\begin{array}{c} (\frac{G^{\prime }}{G})=\{\begin{array}{cc} -\frac{\nu }{2}+\frac{\sqrt{\nu^{2}-4\mu }}{2}\times \frac{A\;\text{cosh}\;(\frac{\sqrt{\nu^{2}-4\mu }}{2}\zeta )+B\;\text{sinh}\;(\frac{\sqrt{\nu^{2}-4\mu }}{2}\zeta )}{A\;\text{sinh}\;(\frac{\sqrt{\nu^{2}-4\mu }}{2}\zeta )+B\;\text{cosh}\;(\frac{\sqrt{\nu^{2}-4\mu }}{2}\zeta )} & \text{if}\;\nu^{2}-4\mu >0\\ -\frac{\nu }{2}+\frac{\sqrt{4\mu -\nu^{2}}}{2}\times \frac{A\;\text{cos}\;(\frac{\sqrt{4\mu -\nu^{2}}}{2}\zeta )-B\;\text{sin}\;(\frac{\sqrt{4\mu -\nu^{2}}}{2}\zeta )}{A\;\text{sin}\;(\frac{\sqrt{4\mu -\nu^{2}}}{2}\zeta )+B\;\text{cos}\;(\frac{\sqrt{4\mu -\nu^{2}}}{2}\zeta )} & \text{if}\;\nu^{2}-4\mu <0\\ -\frac{\nu }{2}+\frac{B}{A+B\zeta } & \text{if}\;\nu^{2}-4\mu =0. \end{array} \end{array}$$ (8)

To estimate the unknown wave function *u*(*x*, *t*) = *V*(*ζ*), we follow the succeeding steps.

Step 1. Establishing homogeneous balance between the highest-order derivative of *V* and the highest-order nonlinear term in Eq (5) allows for the determination of the integer *n*.
Step 2. After determining *n*, the Eqs (6) and (7) are replaced into Eq (5) and then the Eq (5) becomes a polynomial of $\left(\frac{G^{\prime }}{G}\right)$. Equating the coefficients of the same powers of $\left(\frac{G^{\prime }}{G}\right)$, this polynomial yields a set of nonlinear algebraic equations involving *α*_*j*_, *β*_*j*_, *ν*, *μ*, *k*, and *ω*.
Step 3. Solving the derived system for *α*_*j*_, *β*_*j*_, *k*, and *ω*, and subsequently substituting the determined values of *α*_*j*_, *β*_*j*_, along with the general solution of (7) into Eq (6), yield the sought-after solutions for Eq (4).

Although numerous attempts have been made to discover different techniques for solving both integrable and non-integrable NLPDEs, developing a unified method for solving these equations is still difficult to achieve. The rational $\left(\frac{G^{\prime }}{G}\right)$-expansion method excels in generating exact traveling wave solutions for NLPDEs, delivering solutions of high precision and accuracy. These precise solutions play a crucial role in elucidating the fundamental principles underlying physical phenomena. The method we are discussing offers advantages compared to other approaches. The primary advantage of the rational $\left(\frac{G^{\prime }}{G}\right)$-expansion method compared to other methods lie in its provision of more general solutions featuring several free parameters. Through judicious parameter selection, these solutions can coincide with established solutions obtained through existing methods. Again, the proposed technique handles NLPDEs in a direct manner, requiring no initial/boundary conditions or initial trial function at the outset, unlike numerical methods. The capacity of the suggested approach to incorporate a larger number of arbitrary constants compared to other existing techniques renders it especially valuable in the realm of mathematical physics for the analysis of nonlinear wave phenomena.

Despite its numerous benefits, it is important to consider the limitations of the method. This method occasionally generates solutions that resemble well-known solutions obtainable through alternative techniques. Furthermore, depending on the efficiency of the computer, the rational $\left(\frac{G^{\prime }}{G}\right)$-expansion approach may not guarantee the existence of solutions to the resulting algebraic equations when the balance number of the reduced ordinary differential equation exceeds two.

## 3. Analytical wave solutions of the mBBM equation

In this section the rational $\left(\frac{G^{\prime }}{G}\right)$-expansion method is applied on the mBBM Eq (3) to find its analytical wave solutions. The wave variable transformations *u*(*x*, *t*) = *V*(*ζ*), *ζ* = *lx* + *ωt* converts the stated model (3) into the following ODE:

$$\begin{array}{c} \left(\omega +l\alpha \right)V+\frac{l\beta }{3}V^{3}-\omega l^{2}\gamma V^{\prime \prime }=0, \end{array}$$ (9)

where *l* and *ω* represents the wave number and speed of the wave respectively. Balancing the term *V*^3^ with *V*′′ which are present in Eq (9), we get *n* = 1. Then the solution for this equation can be written as

$$\begin{array}{c} V\left(\zeta \right)=\frac{a_{0}+a_{1}(\frac{G^{\prime }}{G})}{b_{0}+b_{1}(\frac{G^{\prime }}{G})}, \end{array}$$ (10)

where, the constants *a*_0_, *a*_1_, *b*_0_, and *b*_1_ are unspecified and will be determined subsequently.

Now using step 2 of the technique and replacing the values of Eqs (10) along with (7) in (9), we obtain the subsequent system of equations:

$$\begin{array}{cc} {\left(\frac{G^{\prime }}{G}\right)}^{3}:\; & -3\gamma l^{2}\nu \omega a_{0}b_{1}^{2}+3\gamma l^{2}\nu \omega a_{1}b_{0}b_{1}+6\gamma l^{2}\omega a_{0}b_{0}b_{1}-6\gamma l^{2}\omega a_{1}b_{0}^{2}+3\alpha la_{1}b_{1}^{2}+\beta la_{1}^{3}\\ & +3\omega a_{1}b_{1}^{2}, \end{array}$$ (11a)

$$\begin{array}{cc} {\left(\frac{G^{\prime }}{G}\right)}^{2}:\; & -3\gamma l^{2}\nu^{2}\omega a_{0}b_{1}^{2}+3\gamma l^{2}\nu^{2}\omega a_{1}b_{0}b_{1}+9\gamma l^{2}\nu \omega a_{0}b_{0}b_{1}-9\gamma l^{2}\nu \omega a_{1}b_{0}^{2}-6\gamma l^{2}\mu \omega a_{0}b_{1}^{2}\\ & +6\gamma l^{2}\mu \omega a_{1}b_{0}b_{1}+3\alpha la_{0}b_{1}^{2}+6\alpha la_{1}b_{0}b_{1}+3\beta la_{0}a_{1}^{2}+3\omega a_{0}b_{1}^{2}+6\omega a_{1}b_{0}b_{1}, \end{array}$$ (11b)

$$\begin{array}{cc} {\left(\frac{G^{\prime }}{G}\right)}^{1}:\; & 3\gamma l^{2}\nu^{2}\omega a_{0}b_{0}b_{1}-3\gamma l^{2}\nu^{2}\omega a_{1}b_{0}^{2}-9\gamma l^{2}\nu \mu \omega a_{0}b_{1}^{2}+9\gamma l^{2}\nu \mu \omega a_{1}b_{0}b_{1}+6\gamma l^{2}\mu \omega a_{0}b_{0}b_{1}\\ & -6\gamma l^{2}\mu \omega a_{1}b_{0}^{2}+6\alpha la_{0}b_{0}b_{1}+3\alpha la_{1}b_{0}^{2}+3\beta la_{0}^{2}a_{1}+6\omega a_{0}b_{0}b_{1}+3\omega a_{1}b_{0}^{2}, \end{array}$$ (11c)

$$\begin{array}{ll} {\left(\frac{G^{\prime }}{G}\right)}^{0} & :3\gamma l^{2}\nu \mu \omega a_{0}b_{0}b_{1}-3\gamma l^{2}\nu \mu \omega a_{1}b_{0}^{2}-6\gamma l^{2}\mu^{2}\omega a_{0}b_{1}^{2}+6\gamma l^{2}\mu^{2}\omega a_{1}b_{0}b_{1}+3\alpha la_{0}b_{0}^{2}+\\ & \beta la_{0}^{3}+3\omega a_{0}b_{0}^{2}. \end{array}$$ (11d)

By solving the above system using Maple, we obtain the following sets of solutions:

Set 1:

$$\begin{array}{cc} & l=l,\omega =-\frac{2\alpha l}{\gamma l^{2}\nu^{2}-4\gamma l^{2}\mu +\;2},a_{0}=\pm \frac{3\gamma l\nu \alpha b_{0}}{\sqrt{-3\alpha \beta \gamma (2+\gamma l^{2}\left(\nu^{2}-4\mu \right))}},\\ & a_{1}=\pm \frac{6\gamma l\alpha b_{0}}{\sqrt{-3\alpha \beta \gamma (2+\gamma l^{2}\left(\nu^{2}-4\mu \right))}},b_{0}=b_{0},b_{1}=0. \end{array}$$ (12)

Set 2:

$$\begin{array}{cc} & l=l,\omega =-\frac{2\alpha l}{\gamma l^{2}\nu^{2}-4\gamma l^{2}\mu +\;2},a_{0}=\pm \sqrt{-\frac{3\alpha \gamma }{4\beta (2+\gamma l^{2}(\nu^{2}-4\mu ))}}(\nu^{2}-4\mu )lb_{1},\\ & a_{1}=0,b_{0}=\frac{b_{1}\nu }{2},b_{1}=b_{1}. \end{array}$$ (13)

Set 3:

$$\begin{array}{cc} & l=l,\omega =-\frac{2\alpha l}{\gamma l^{2}\nu^{2}-4\gamma l^{2}\mu +\;2},a_{0}=\pm \sqrt{-\frac{3\alpha \gamma }{\beta (2+\gamma l^{2}(\nu^{2}-4\mu ))}}(-\nu b_{0}+2\mu b_{1})l,\\ & a_{1}=\pm \sqrt{-\frac{3\alpha \gamma }{\beta (2+\gamma l^{2}(\nu^{2}-4\mu ))}}(\nu b_{1}-2b_{0})l,b_{0}=b_{0},b_{1}=b_{1}. \end{array}$$ (14)

Set 4:

$$\begin{array}{cc} & l=\pm \frac{\nu }{\sqrt{-2\gamma \mu (\nu^{2}-4\mu )}},\omega =\frac{4\nu \alpha \mu }{\left(\nu^{2}-4\mu \right)\sqrt{-2\gamma \mu (\nu^{2}-4\mu )}},a_{0}=\pm \frac{b_{1}\nu }{2}\sqrt{-\frac{3\alpha }{\beta }},a_{1}=0,\\ & b_{0}=\frac{b_{1}\nu }{2},b_{1}=b_{1}. \end{array}$$ (15)

Substituting the provided estimates from (12)–(15) into the solution (10), we get

$$\begin{array}{c} V_{1}(\zeta )=\pm \frac{3\gamma \alpha l(2(\frac{G^{\prime }}{G})+\nu )}{\sqrt{-3\alpha \beta \gamma (2+\gamma l^{2}(\nu^{2}-4\mu ))}}, \end{array}$$ (16)

where $\zeta =lx-\frac{2\alpha lt}{\gamma l^{2}(\nu^{2}-4\mu )+2}$.

$$\begin{array}{c} V_{2}\left(\zeta \right)=\pm \frac{\sqrt{-\frac{3\alpha \gamma }{\beta (2+\gamma l^{2}(\nu^{2}-4\mu ))}}(\nu^{2}-4\mu )l}{2(\frac{G^{\prime }}{G})+\nu }, \end{array}$$ (17)

where $\zeta =lx-\frac{2\alpha lt}{\gamma l^{2}(\nu^{2}-4\mu )+2}$.

$$\begin{array}{c} V_{3}(\zeta )=\pm \frac{l\sqrt{-\frac{3\alpha \gamma }{\beta (2+\gamma l^{2}(\nu^{2}-4\mu ))}}(-\nu b_{0}+2\mu b_{1}+\left(\nu b_{1}-2b_{0}\right)(\frac{G^{\prime }}{G}))}{b_{0}+b_{1}(\frac{G^{\prime }}{G})}, \end{array}$$ (18)

where $\zeta =lx-\frac{2\alpha lt}{\gamma l^{2}(\nu^{2}-4\mu )+2}$.

$$\begin{array}{c} V_{4}(\zeta )=\pm \frac{\sqrt{-\frac{3\alpha }{\beta }}\nu }{2(\frac{G^{\prime }}{G})+\nu }, \end{array}$$ (19)

where $\zeta =\frac{\nu }{\sqrt{-2\gamma \mu (\nu^{2}-4\mu )}}(\pm x+\frac{4\alpha \mu t}{\nu^{2}-4\mu })$.

The traveling wave solutions for the mBBM model (3) are derived by employing the outcomes presented in (8) based on the solution (16) in the following manner:

When *ν*^2^ − 4*μ* > 0, the hyperbolic trigonometric solution is obtained as

$$\begin{array}{c} V_{11}\left(\zeta \right)=\pm \frac{3\gamma \alpha l\sqrt{\nu^{2}-4\mu }\;}{\sqrt{-3\alpha \beta \gamma (2+\gamma l^{2}(\nu^{2}-4\mu ))}}(\frac{A\text{cosh}\;(\frac{\sqrt{\nu^{2}-4\mu }}{2}\zeta )\;+B\text{sinh}\;(\frac{\sqrt{\nu^{2}-4\mu }}{2}\zeta )\;}{A\text{sinh}\;(\frac{\sqrt{\nu^{2}-4\mu }}{2}\zeta )\;+B\text{cosh}\;(\frac{\sqrt{\nu^{2}-4\mu }}{2}\zeta )\;}), \end{array}$$ (20)

where *A*, *B* are integral constants and $\zeta =lx-\frac{2\alpha lt}{\gamma l^{2}(\nu^{2}-4\mu )+2}$.

When *ν*^2^ − 4*μ* < 0, the trigonometric solution is achieved as

$$\begin{array}{c} V_{12}(\zeta )=\pm \frac{3\gamma \alpha l\sqrt{4\mu -\nu^{2}}}{\sqrt{-3\alpha \beta \gamma (2+\gamma l^{2}(\nu^{2}-4\mu ))}}(\frac{A\text{cos}\;(\frac{\sqrt{4\mu -\nu^{2}}}{2}\zeta )\;-B\text{sin}\;(\frac{\sqrt{4\mu -\nu^{2}}}{2}\zeta )\;}{A\text{sin}\;(\frac{\sqrt{4\mu -\nu^{2}}}{2}\zeta )\;+B\text{cos}\;(\frac{\sqrt{4\mu -\nu^{2}}}{2}\zeta )\;}), \end{array}$$ (21)

where *A*, *B* are arbitrary constants and $\zeta =lx-\frac{2\alpha lt}{\gamma l^{2}(\nu^{2}-4\mu )+2}$.

When *ν*^2^ − 4*μ* = 0, the rational solution is gained as

$$\begin{array}{c} V_{13}\left(\zeta \right)=\pm \frac{3\gamma \alpha l\;}{\sqrt{-6\alpha \beta \gamma }}(\frac{2B}{A+B\zeta }), \end{array}$$ (22)

where *A*, *B* are free constants and *ζ* = *l*(*x* − *αt*).

Incorporating the solutions organized in Eq (8) into Eq (17), we obtain subsequent soliton evaluations for the mBBM Eq (3):

For *ν*^2^ − 4*μ* > 0, the hyperbolic trigonometric solution is obtained as

$$\begin{array}{c} V_{21}\left(\zeta \right)=\pm l\sqrt{-\frac{3\alpha \gamma (\nu^{2}-4\mu )}{\beta (2+\gamma l^{2}(\nu^{2}-4\mu ))}}(\frac{A\text{sinh}\;(\frac{\sqrt{\nu^{2}-4\mu }}{2}\zeta )\;+B\text{cosh}\;(\frac{\sqrt{\nu^{2}-4\mu }}{2}\zeta )\;}{A\text{cosh}\;(\frac{\sqrt{\nu^{2}-4\mu }}{2}\zeta )\;+B\text{sinh}\;(\frac{\sqrt{\nu^{2}-4\mu }}{2}\zeta )\;}), \end{array}$$ (23)

and for *ν*^2^ − 4*μ* < 0, the trigonometric solution is achieved as

$$\begin{array}{c} V_{22}\left(\zeta \right)=\pm l\sqrt{-\frac{3\alpha \gamma (4\mu -\nu^{2})}{\beta (2+\gamma l^{2}(\nu^{2}-4\mu ))}}(\frac{A\text{sin}\;(\frac{\sqrt{4\mu -\nu^{2}}}{2}\zeta )\;+B\text{cos}\;(\frac{\sqrt{4\mu -\nu^{2}}}{2}\zeta )\;}{A\text{cos}\;(\frac{\sqrt{4\mu -\nu^{2}}}{2}\zeta )\;-B\text{sin}\;(\frac{\sqrt{4\mu -\nu^{2}}}{2}\zeta )\;}), \end{array}$$ (24)

where *A*, *B* are random constants and $\zeta =lx-\frac{2\alpha lt}{\gamma l^{2}(\nu^{2}-4\mu )+2}$.

By employing the solutions provided in (8) within the context of (18), we derive the subsequent progressive wave solutions for the mBBM Eq (3):

If *ν*^2^ − 4*μ* > 0, the hyperbolic trigonometric solution is simplified as

$$\begin{array}{cc} & V_{31}\left(\zeta \right)=\pm l\sqrt{-\frac{3\alpha \gamma }{\beta (2+\gamma l^{2}\left(\nu^{2}-4\mu \right))}}\times \\ & \;(-\nu b_{0}+2\mu b_{1}+\left(\nu b_{1}-2b_{0}\right)(-\frac{\nu }{2}+\frac{\sqrt{\nu^{2}-4\mu }}{2}\times \frac{A\;\text{cosh}\;(\frac{\sqrt{\nu^{2}-4\mu }}{2}\zeta )+B\;\text{sinh}\;(\frac{\sqrt{\nu^{2}-4\mu }}{2}\zeta )}{A\;\text{sinh}\;(\frac{\sqrt{\nu^{2}-4\mu }}{2}\zeta )+B\;\text{cosh}\;(\frac{\sqrt{\nu^{2}-4\mu }}{2}\zeta )}))\\ & \;\times {\left(b_{0}+b_{1}(-\frac{\nu }{2}+\frac{\sqrt{\nu^{2}-4\mu }}{2}\times \frac{A\;\text{cosh}\;(\frac{\sqrt{\nu^{2}-4\mu }}{2}\zeta )+B\;\text{sinh}\;(\frac{\sqrt{\nu^{2}-4\mu }}{2}\zeta )}{A\;\text{sinh}\;(\frac{\sqrt{\nu^{2}-4\mu }}{2}\zeta )+B\;\text{cosh}\;(\frac{\sqrt{\nu^{2}-4\mu }}{2}\zeta )})\right)}^{-1}, \end{array}$$ (25)

If *ν*^2^ − 4*μ* < 0, the trigonometric solution is calculated as

$$\begin{array}{cc} & V_{32}(\zeta )=\pm l\sqrt{-\frac{3\alpha \gamma }{\beta (2+\gamma l^{2}(\nu^{2}-4\mu ))}}\\ & \;(-\nu b_{0}+2\mu b_{1}+\left(\nu b_{1}-2b_{0}\right)(-\frac{\nu }{2}+\frac{\sqrt{4\mu -\nu^{2}}}{2}\times \frac{A\text{cos}\;(\frac{\sqrt{4\mu -\nu^{2}}}{2}\zeta )\;-B\text{sin}\;(\frac{\sqrt{4\mu -\nu^{2}}}{2}\zeta )\;}{A\text{sin}\;(\frac{\sqrt{4\mu -\nu^{2}}}{2}\zeta )\;+B\text{cos}\;(\frac{\sqrt{4\mu -\nu^{2}}}{2}\zeta )\;}))\\ & \;\times {\left(b_{0}+b_{1}(-\frac{\nu }{2}+\frac{\sqrt{4\mu -\nu^{2}}}{2}\times \frac{A\text{cos}\;(\frac{\sqrt{4\mu -\nu^{2}}}{2}\zeta )\;-B\text{sin}\;(\frac{\sqrt{4\mu -\nu^{2}}}{2}\zeta )\;}{A\text{sin}\;(\frac{\sqrt{4\mu -\nu^{2}}}{2}\zeta )\;+B\text{cos}\;(\frac{\sqrt{4\mu -\nu^{2}}}{2}\zeta )\;})\right)}^{-1}, \end{array}$$ (26)

If *ν*^2^ − 4*μ* = 0, the rational solution is simplified as

$$\begin{array}{c} V_{33}(\zeta )=\pm \frac{l\sqrt{-\frac{3\alpha \gamma }{2\beta }}(-\nu b_{0}+2\mu b_{1}+\left(\nu b_{1}-2b_{0}\right)(-\frac{\nu }{2}+\frac{B}{A+B\zeta }))}{b_{0}+b_{1}(-\frac{\nu }{2}+\frac{B}{A+B\zeta })}, \end{array}$$ (27)

where *A*, *B* are subjective constants and $\zeta =lx-\frac{2\alpha lt}{\gamma l^{2}(\nu^{2}-4\mu )+2}$.

Lastly, by utilizing the solutions compiled in (8) based on (18), we ascertain the subsequent exact wave solutions for the mBBM Eq (3):

Because of *ν*^2^ − 4*μ* > 0, the hyperbolic function solution is ascertained as

$$\begin{array}{c} V_{41}(\zeta )=\pm \nu \sqrt{-\frac{3\alpha }{\beta (\nu^{2}-4\mu )}}(\frac{A\text{sinh}\;(\frac{\sqrt{\nu^{2}-4\mu }}{2}\zeta )\;+B\text{cosh}\;(\frac{\sqrt{\nu^{2}-4\mu }}{2}\zeta )\;}{A\text{cosh}\;(\frac{\sqrt{\nu^{2}-4\mu }}{2}\zeta )\;+B\text{sinh}\;(\frac{\sqrt{\nu^{2}-4\mu }}{2}\zeta )\;}), \end{array}$$ (28)

Because of *ν*^2^ − 4*μ* < 0, the trigonometric function solution is attained as

$$\begin{array}{c} V_{42}(\zeta )=\pm \nu \sqrt{-\frac{3\alpha }{\beta (4\mu -\nu^{2})}}(\frac{A\text{sin}\;(\frac{\sqrt{4\mu -\nu^{2}}}{2}\zeta )\;+B\text{cos}\;(\frac{\sqrt{4\mu -\nu^{2}}}{2}\zeta )\;}{A\text{cos}\;(\frac{\sqrt{4\mu -\nu^{2}}}{2}\zeta )\;-B\text{sin}\;(\frac{\sqrt{4\mu -\nu^{2}}}{2}\zeta )\;}), \end{array}$$ (29)

where *A*, *B* are any constants and $\zeta =\frac{\nu }{\sqrt{-2\gamma \mu (\nu^{2}-4\mu )}}(\pm x+\frac{4\alpha \mu t}{\nu^{2}-4\mu })$.

## 4. Characteristics of the wave solutions

In this section, we delve into the attributes of certain solutions identified from the mBBM model. The rational $\left(\frac{G^{\prime }}{G}\right)$-expansion method was utilized for the mBBM equation, yielding wave solutions characterized by various free parameters. This section explores the graphical representations and explanations of these solutions in the context of the mBBM equation. Various 3D, density and 2D plots illustrating the obtained solutions are generated using Matlab and Maple software, allowing for a comprehensive analysis of their nature. To emphasize this point, by assigning different values to the free parameters we have generated plots depicting the solutions (20), (21), (22), (23), (25), (27), (28) and (29). This exploration showcases various types of solitons, including kink, anti-kink, anti-peakon, singular periodic, anti-bell (dark) and V-shaped solitons. Different combinations of the free parameters utilized in the study may result in the emergence of diverse soliton solutions.

**Fig 1:**
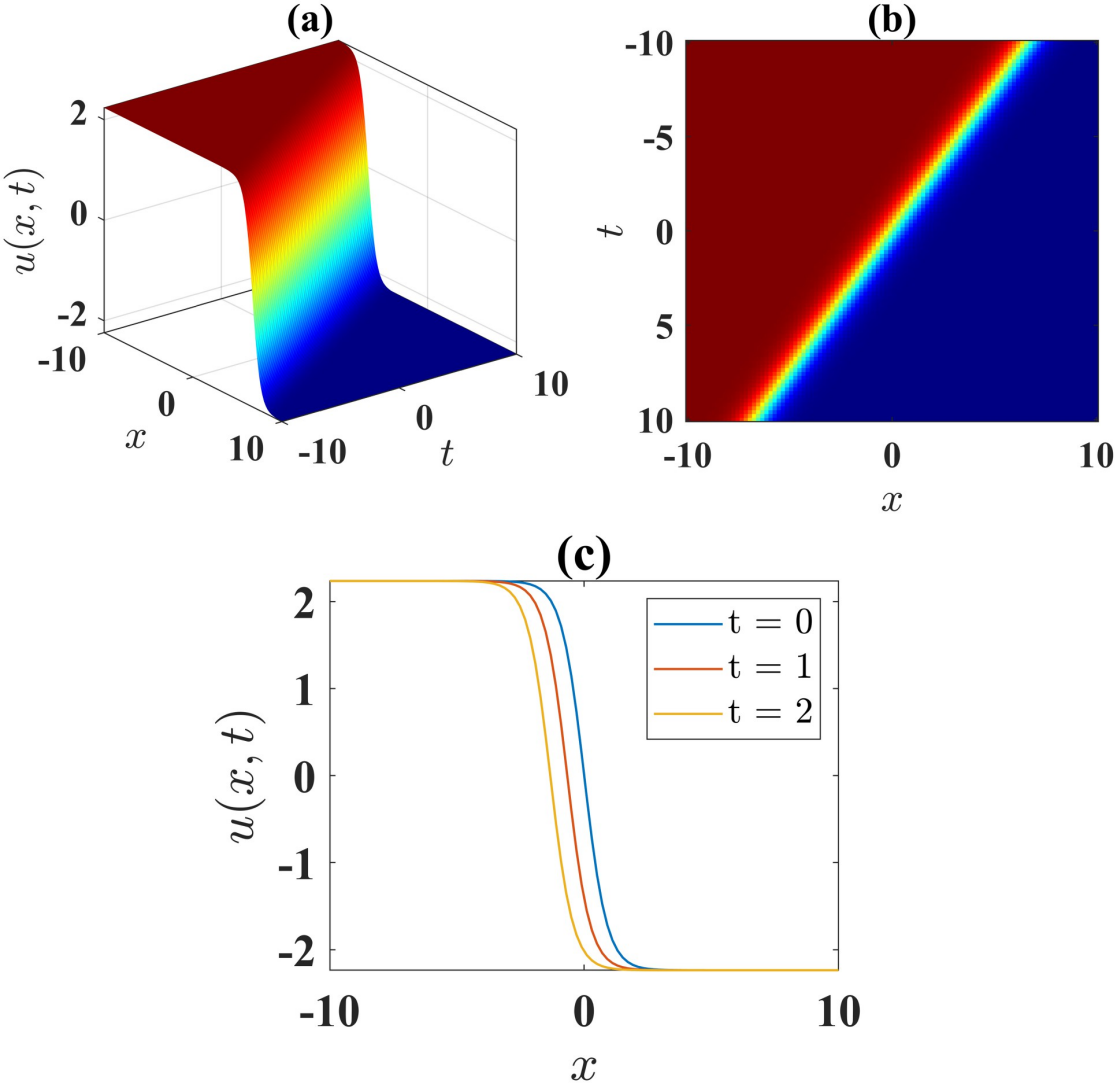
(a) 3D surface plot, (b) density plot and (c) 2D combined plots of (20), when *α* = 1, *β* = −1, *l* = 1, *γ* = −1, *ν* = 1, *μ* = −1, *A* = 0, *B* = 1.

The solution (20) describes a soliton with anti-kink shape, where parameters *α* = 1, *β* = −1, *l* = 1, *γ* = −1, *ν* = 1, *μ* = −1, *A* = 0 and *B* = 1 are employed. This soliton is illustrated in Fig 1 for the limit −10 ≤ *x*, *t* ≤ 10. Fig 1(b) illustrates the contour plot of the solution (20), while Fig 1(c) presents the 2D plots at different time levels.

**Fig 2:**
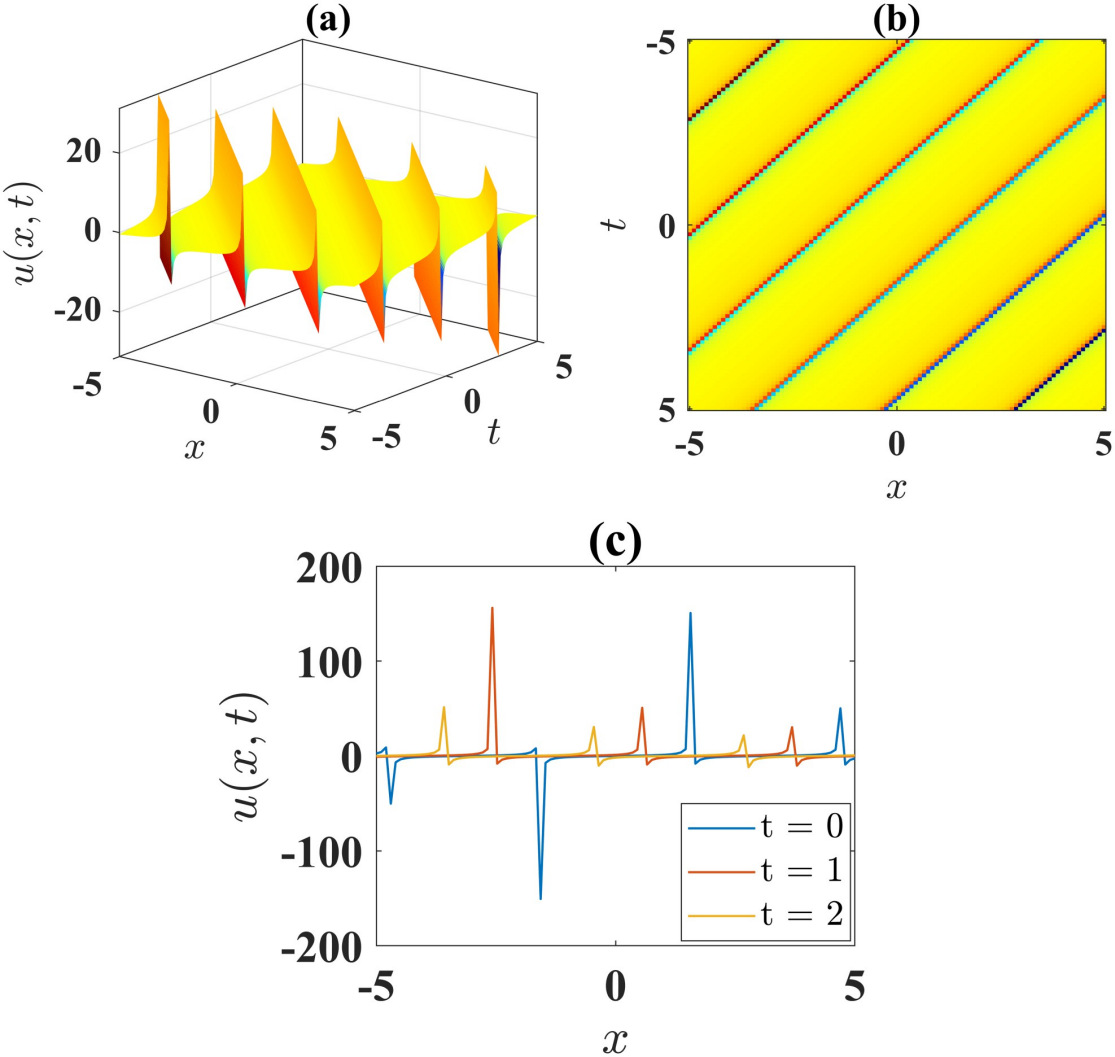
(a) 3D surface plot, (b) density plot and (c) 2D combined plots of (21), when *α* = 1, *β* = 10, *l* = 1, *γ* = 1, *ν* = 4, *μ* = 5, *A* = 0 and *B* = 1.

The expression (21) represents a singular periodic soliton observed for parameters *α* = 1, *β* = 10, *l* = 1, *γ* = 1, *ν* = 4, *μ* = 5, *A* = 0 and *B* = 1 within −5 ≤ *x*, *t* ≤ 5, as shown in Fig 2. Fig 2(b) illustrates the contour plot of the solution (20), while Fig 2(c) presents the 2D plots at different time levels.

**Fig 3:**
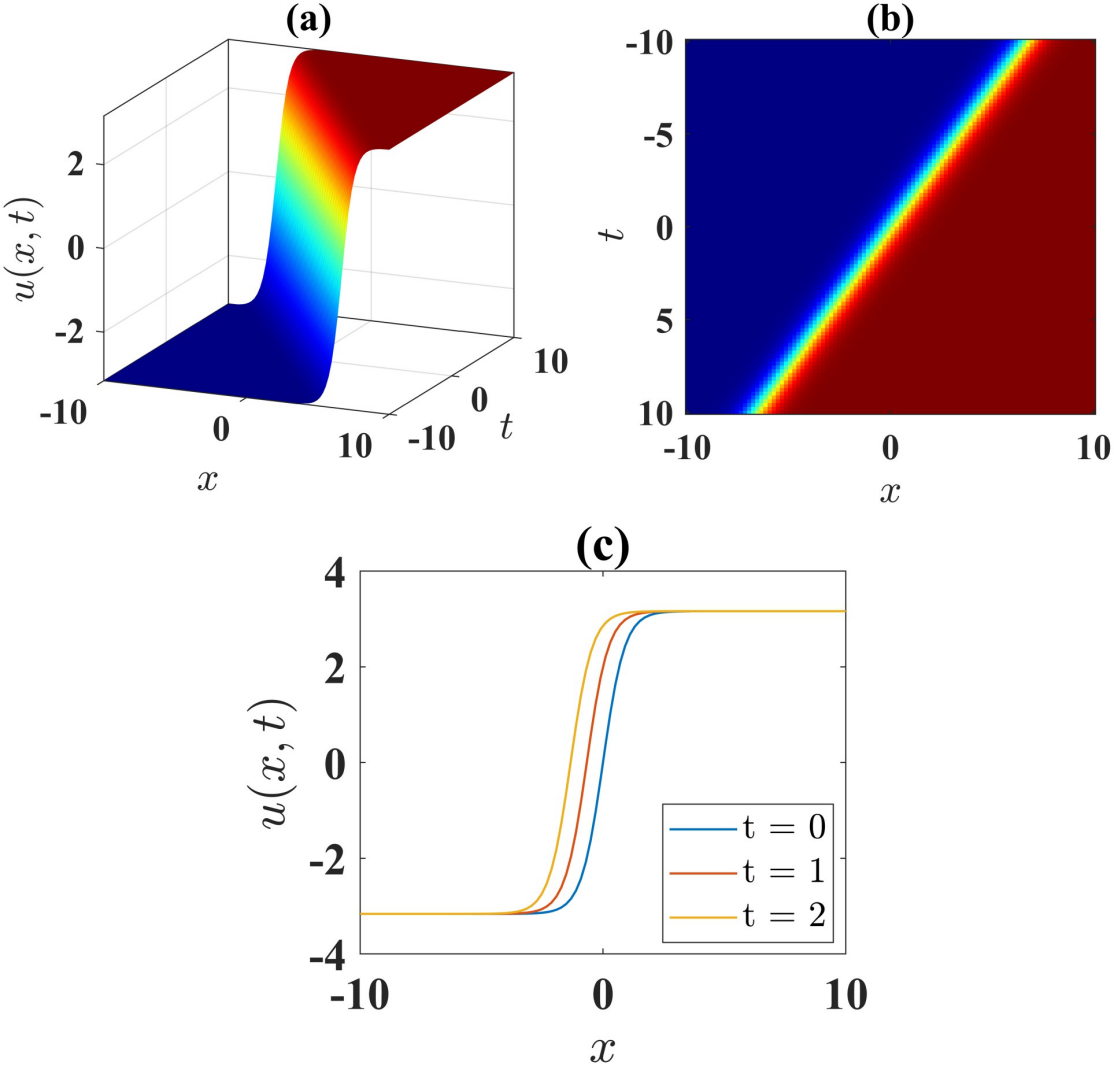
(a) 3D surface plot, (b) density plot and (c) 2D combined plots of (23), when *α* = 1, *β* = −0.5, *l* = 1, *γ* = −1, *ν* = 1, *μ* = −1, *A* = 1, *B* = 0.

The expression (23) describes the features of the kink soliton when *α* = 1, *β* = −0.5, *l* = 1, *γ* = −1, *ν* = 1, *μ* = −1, *A* = 1 and *B* = 0 over the interval −10 ≤ *x*, *t* ≤ 10, as exhibited in Fig 3. Fig 3(b) illustrates the contour plot of the solution (20), while Fig 3(c) presents the 2D plots at different time levels.

**Fig 4:**
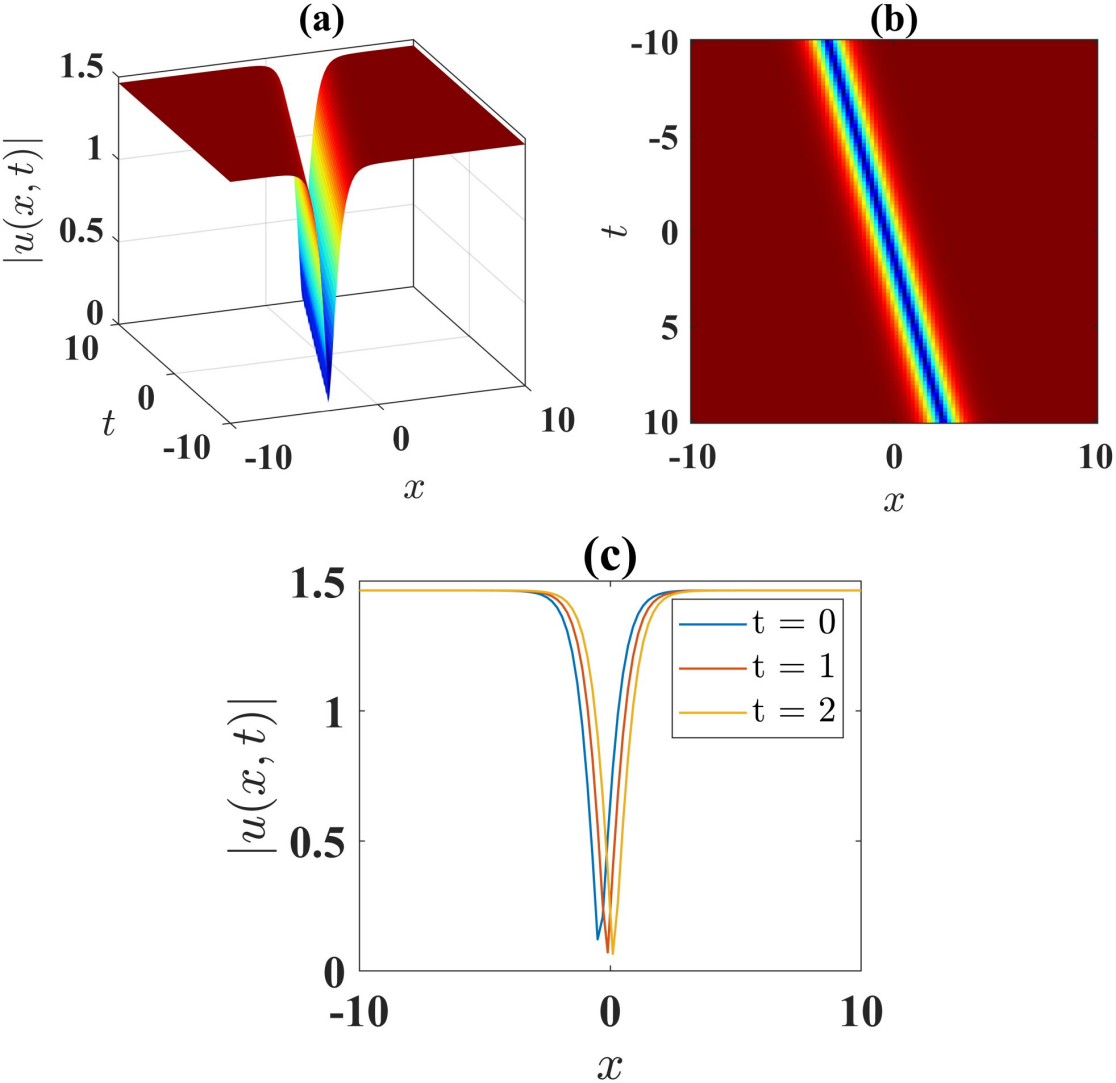
(a) 3D surface plot, (b) density plot and (c) 2D combined plots of (25), when *α* = 1, *β* = −0.5, *l* = 1, *γ* = −1, *ν* = 1, *μ* = −1, *A* = 1, *B* = 0.

The dark soliton, manifested in Fig 4, is expressed by the absolute value of the solution (25) with parameters *α* = 1, *β* = 1, *l* = 1, *γ* = 1, *ν* = 1, *μ* = −1, *b*_0_ = 1, *b*_1_ = 1, *A* = 1 and *B* = 0, confined within the boundaries −5 ≤ *x*, *t* ≤ 5. Fig 4(b) illustrates the contour plot of the solution (20), while Fig 4(c) presents the 2D plots at different time levels.

**Fig 5:**
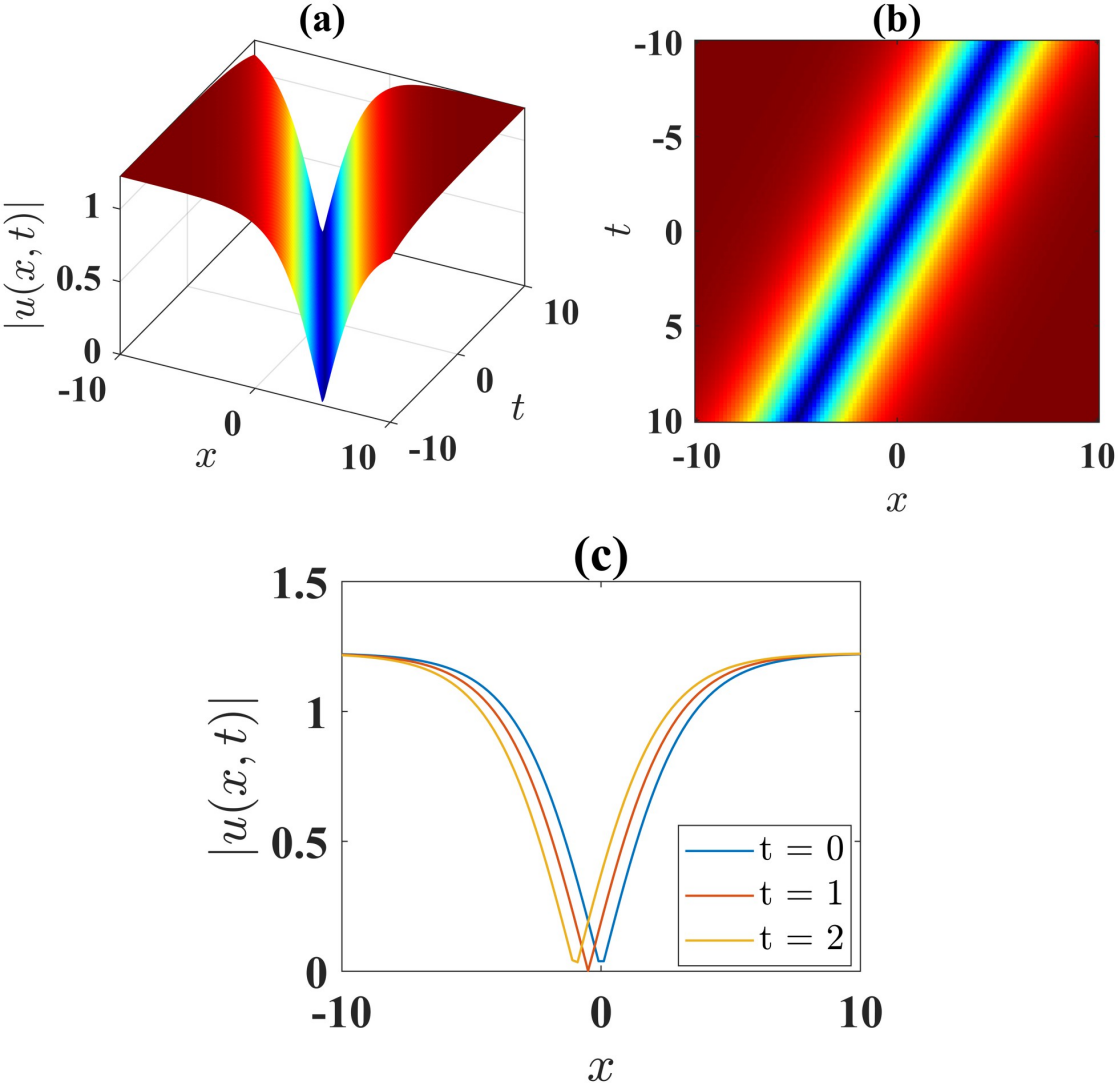
(a) 3D surface plot, (b) density plot and (c) 2D combined plots of (28), when *α* = 1, *β* = −0.5, *l* = 1, *γ* = −1, *ν* = 1, *μ* = −1, *A* = 1, *B* = 0.

In Fig 5, it is evident that the modulus of the solution (28) produces an anti-peakon soliton for the given parameters *α* = 1, *β* = 1, *γ* = 5, *ν* = 2, *μ* = −1, *A* = 1 and *B* = 0, within the specified range of −10 ≤ *x*, *t* ≤ 10. Fig 5(b) illustrates the contour plot of the solution (20), while Fig 5(c) presents the 2D plots at different time levels.

**Fig 6:**
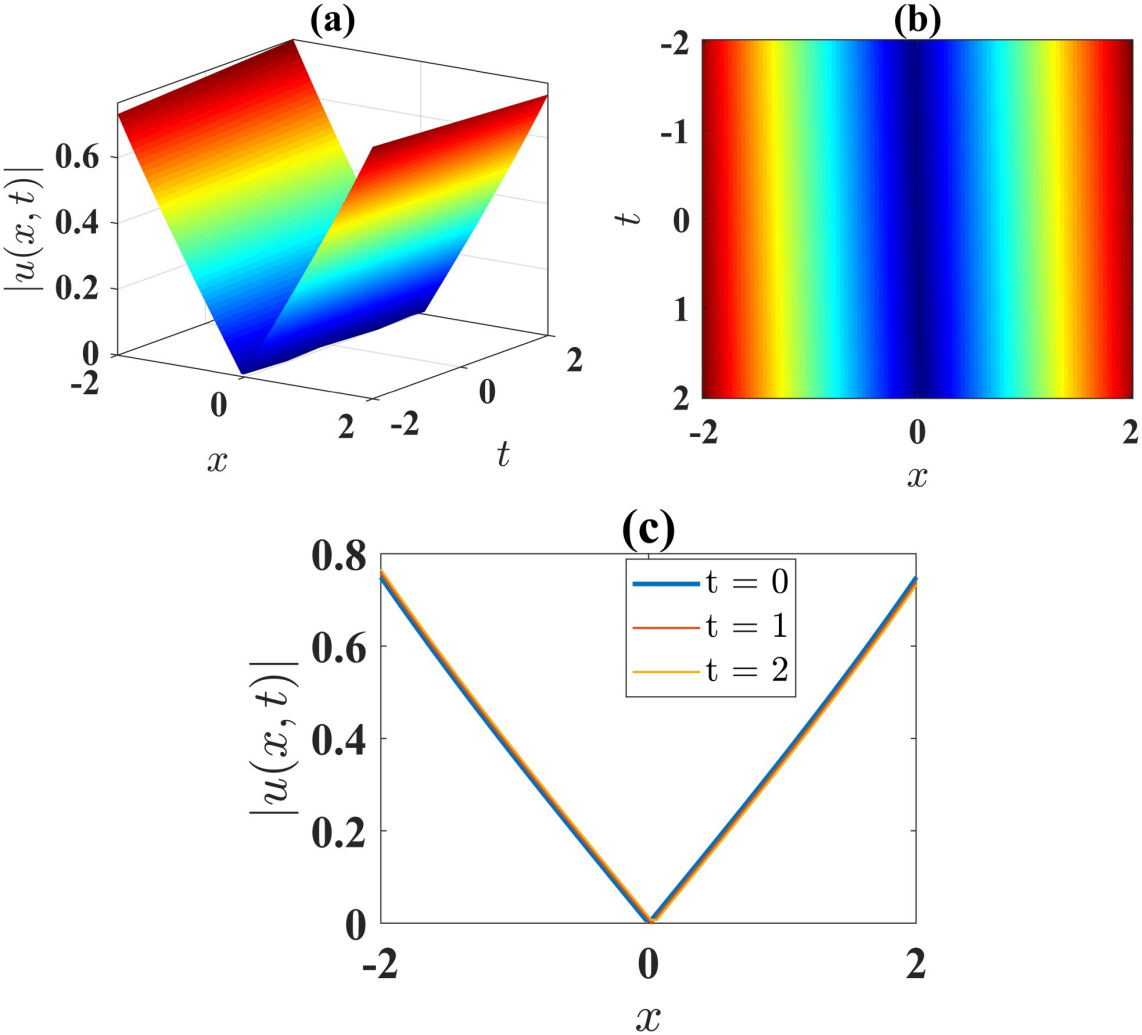
(a) 3D surface plot, (b) density plot and (c) 2D combined plots of (29), when *α* = −0.01, *β* = −0.01, *γ* = 6, *ν* = 2, *μ* = 2, *A* = 1, *B* = 0.

The V-shaped soliton, as depicted in Fig 6, is obtained from the absolute value of the solution (29) under the conditions *α* = −0.01, *β* = −0.01, *γ* = 6, *ν* = 2, *μ* = 2, *A* = 1 and *B* = 0. This soliton is observed within the domain −2 ≤ *x*, *t* ≤ 2. Fig 6(b) illustrates the contour plot of the solution (20), while Fig 6(c) presents the 2D plots at different time levels.

The analysis presented above demonstrates the effectiveness of the described method in generating accurate wave solutions for the mBBM model, encompassing diverse profiles. Among various wave structures, kink-type solitons are traveling wave solutions that transition from one phase to another by rising or descending. As they propagate to infinity, kink solitons eventually stabilize at a constant value. Additionally, kink-type solitons play a vital role in various fields such as nonlinear optics and condensed matter physics. They represent stable, localized transitions between distinct states within materials, offering valuable insights into defects in crystalline structures, phase transitions, and optical communication systems.

A periodic soliton is a robustly formed solitary wave capable of traveling long distances with consistent velocity and intensity. It maintains a clearly defined amplitude and phase, representing a highly stable solution. Such solitons are employed for transmitting signals across long distances.

Singular solitons are significant for understanding the mathematical modeling of physical problems, but they have limited applicability in practical physical scenarios. These are a form of solitary wave that has a singularity, which is typically an infinite discontinuity. Singularities can appear practically everywhere, and they are surprisingly common in the mathematical models that physicists employ to study the universe. Furthermore, singular solitons denote sudden, concentrated disturbances within physical systems, playing a crucial role in diverse fields such as nonlinear optics and fluid dynamics. They embody localized regions of intense energy or complex structures, providing insights into intricate phenomena, shockwaves, and compact information carriers within nonlinear dynamic wave equations [45].

Dark solitons are dark pulses that exist within the interior of a continuous wave, maintaining a distinct structure while preserving their propagation characteristics. They are pulses that propagate through various media such as liquids, gases, plasmas, and solids.

An anti-peakon soliton is characterized by a discontinuous first derivative at its lowest peak point. Similar to the kink soliton, an anti-peakon soliton maintains a constant value as time approaches infinity. It is also known as dark soliton in optical fiber studies.

V-shaped dark solitons offer soliton control in optical fiber studies. They suddenly change direction at the zero point, maintaining the original pulse duration and velocity. This behavior may have applications in the design of all optical switches. Therefore, the importance and essential role of different solitons become undeniable in elucidating nonlinear and intricate physical phenomena.

## 5. Comparison and stability analysis

### 5.1 Comparison

In this su-bsection, we have compared the solutions derived in this article with those documented in the existing literature for the mBBM equation. Many investigations in existing literature have explored the analysis of the mBBM equation employing various methodologies. Specifically, we contrasted the solutions obtained through the $\left(\frac{G^{\prime }}{G}\right)$ technique [32] and the Sardar-subequation method [43] with the outcomes presented in this work. The $\left(\frac{G^{\prime }}{G}\right)$ method and the Sardar-subequation method are used to analyze the NLPDE in mathematical physics, each of which possesses distinct characteristics and advantages.

i. Aslan [32] employed the $\left(\frac{G^{\prime }}{G}\right)$-expansion method to discover exact and explicit solutions for (3). He had successfully derived one set of solutions for the mBBM model. In our approach, when *b*_0_ = 1 and *b*_1_ = 0, it reduces to the $\left(\frac{G^{\prime }}{G}\right)$-expansion method. Notably, we have identified four distinct set of solutions using this modified method. Among these sets, the solutions in Set 1 perfectly align with all the solutions previously derived in [32]. Consequently, it can be asserted that the rational $\left(\frac{G^{\prime }}{G}\right)$ method exhibits greater efficacy compared to the conventional $\left(\frac{G^{\prime }}{G}\right)$-expansion method. Table 1 presents a comparison between the solutions obtained by Aslan [32] and our acquired solutions for the mBBM equation. From the table, it is evident that the obtained solutions *V*_11_(*ζ*), *V*_12_(*ζ*), and *V*_13_(*ζ*) are same as the solutions presented by Aslan [32]. Therefore, the additional solutions for the mBBM equation, derived using the aforementioned method, are all new and significant.
ii. The mBBM model (3), with *α* = 1, *β* = *μ*, and *γ* = −1, was investigated by Naeem et al. [43] using the Sardar-sub equation method to obtain diverse wave solutions. They identified exponential function solutions, which subsequently transformed into hyperbolic and trigonometric solutions. In contrast, our method yields hyperbolic, trigonometric, and rational function solutions. Moreover, our approach involves more parameters than theirs. Describing wave structures becomes more comfortable with solutions containing a greater number of parameters. Therefore, we assert that our method is more successful than theirs in analyzing the mBBM model. Table 2 presents a correlation between the solutions derived by Naeem et al. [43] and our obtained solutions for the mBBM model. It becomes evident that some of the acquired solutions align with those from previous studies under suitable configurations of arbitrary parameters, while others are novel and standard.

**Table 1:** Comparison between Aslan [32] solutions and our obtained solutions for the mBBM equation.

Results of Aslan [32]	Results obtained in this article
For $l(x-\frac{2\alpha }{2+\gamma a}t)=\zeta$, solutions (33), (34) and (35) become: $u\left(x,t\right)=\pm \sqrt{-\frac{3\alpha \gamma a}{\beta (2+\gamma l^{2}a)}}(\frac{A\text{cosh}\;(\frac{\sqrt{a}}{2}\zeta )\;+B\text{sinh}\;(\frac{\sqrt{a}}{2}\zeta )\;}{A\text{sinh}\;(\frac{\sqrt{a}}{2}\zeta )\;+B\text{cosh}\;(\frac{\sqrt{a}}{2}\zeta )\;})$ $u\left(x,t\right)=\pm \sqrt{\frac{3\alpha \gamma a}{\beta (2+\gamma l^{2}a)}}(\frac{A\text{cos}\;(\frac{\sqrt{-a}}{2}\zeta )\;-B\text{sin}\;(\frac{\sqrt{-a}}{2}\zeta )\;}{A\text{sin}\;(\frac{\sqrt{-a}}{2}\zeta )\;+B\text{cos}\;(\frac{\sqrt{-a}}{2}\zeta )\;})$ $u\left(x,t\right)=\pm \frac{\sqrt{6\alpha \gamma }lC_{1}}{\sqrt{-\beta }\left(C_{2}+C_{1}\zeta \right)}$	For *l*^2^(*ν*^2^ − 4*μ*) = *a*, solutions (20), (21) and (22) become: $V_{11}\left(\zeta \right)=\pm \sqrt{-\frac{3\alpha \gamma a}{\beta (2+\gamma l^{2}a)}}(\frac{A\text{cosh}\;(\frac{\sqrt{a}}{2}\zeta )\;+B\text{sinh}\;(\frac{\sqrt{a}}{2}\zeta )\;}{A\text{sinh}\;(\frac{\sqrt{a}}{2}\zeta )\;+B\text{cosh}\;(\frac{\sqrt{a}}{2}\zeta )\;})$ $V_{12}\left(\zeta \right)=\pm \sqrt{\frac{3\alpha \gamma a}{\beta (2+\gamma l^{2}a)}}(\frac{A\text{cos}\;(\frac{\sqrt{-a}}{2}\zeta )\;-B\text{sin}\;(\frac{\sqrt{-a}}{2}\zeta )\;}{A\text{sin}\;(\frac{\sqrt{-a}}{2}\zeta )\;+B\text{cos}\;(\frac{\sqrt{-a}}{2}\zeta )\;})$ $V_{13}\left(\zeta \right)=\pm \frac{\sqrt{6\alpha \gamma }lB}{\sqrt{-\beta }\left(A+B\zeta \right)}$

**Table 2:** Comparison between Naeem et al. [43] solutions and our results for the mBBM equation.

Results of Naeem et al. [43]	Results obtained in this article
For *x* + λ*t* = *ζ*, *p* = 1, *q* = 1, the solutions (28) and (29) may be reduced to: $p\left(x,t\right)=\pm \sqrt{\frac{6}{\mu (1+F)}}(\sqrt{-\frac{F}{2}}\;\text{tanh}\;(\sqrt{-\frac{F}{2}}\zeta ))$ $p\left(x,t\right)=\pm \sqrt{\frac{6}{\mu (1+F)}}(\sqrt{-\frac{F}{2}}\;\text{coth}\;(\sqrt{-\frac{F}{2}}\zeta ))$	For *l*^2^(*ν*^2^ − 4*μ*) = *F*, the solution (20) becomes: $V_{11}\left(\zeta \right)=\pm \sqrt{-\frac{3\alpha \gamma F}{\beta (2+\gamma l^{2}F)}}(\text{tanh}\;(\frac{\sqrt{F}}{2}\zeta ))$; *A* = 0, *B* ≠ 0. $V_{11}\left(\zeta \right)=\pm \sqrt{-\frac{3\alpha \gamma F}{\beta (2+\gamma l^{2}F)}}(\text{coth}\;(\frac{\sqrt{F}}{2}\zeta ))$; *A* ≠ 0, *B* = 0.
For *x* + λ*t* = *ζ*, *p* = 1, *q* = 1, the solutions (33) and (34) may be simplified as: $p\left(x,t\right)=\pm \sqrt{\frac{6}{\mu F+\mu }}(\sqrt{\frac{F}{2}}\;\text{tan}\;(\sqrt{\frac{F}{2}}\zeta ))$ $p\left(x,t\right)=\pm \sqrt{\frac{6}{\mu F+\mu }}(\sqrt{\frac{F}{2}}\;\text{cot}\;(\sqrt{\frac{F}{2}}\zeta ))$	For *l*^2^(*ν*^2^ − 4*μ*) = *F*, the solution (21) can be written as: $V_{12}\left(\zeta \right)=\pm \sqrt{\frac{3\alpha \gamma F}{\beta (2+\gamma l^{2}F)}}(\text{tan}\;(\frac{\sqrt{-F}}{2}\zeta ))$; *A* = 0, *B* ≠ 0. $V_{12}\left(\zeta \right)=\pm \sqrt{\frac{3\alpha \gamma F}{\beta (2+\gamma l^{2}F)}}(\text{cot}\;(\frac{\sqrt{-F}}{2}\zeta ))$; *A* ≠ 0, *B* = 0.

### 5.2 Stability analysis

Numerous nonlinear phenomena exhibit instability, leading to the modulation of steady states due to the interplay between nonlinear and dispersive effects [46]. In this section, we explore the modulation instability of the mBBM Eq (3) through standard linear stability analysis [46, 47]. The linear stability begins with a given steady state solution along with its associated small perturbation, formulated as follows [47, 48]:

$$\begin{array}{c} u\left(x,t\right)=\delta w\left(x,t\right)+H_{0}, \end{array}$$ (30)

where *δ* << 1 denotes the magnitude of the perturbation, and it is evident that any constant value *H*_0_ serves as a steady-state solution to the Eq (3). By introducing the perturbation *δw*(*x*, *t*), we aim to analyze the stability of the steady state under small disturbances. Substituting the solution (30) into (3) and then linearizing in *δ*, we have

$$\begin{array}{c} \delta w_{t}+\alpha \delta w_{x}+\beta \delta H_{0}^{2}w_{x}-\gamma \delta w_{xxt}=0. \end{array}$$ (31)

Eq (31) is a linear constant coefficient partial differential equation with unknown *w*(*x*, *t*). Contemplate a solution to this equation in the following form [47]:

$$\begin{array}{c} w=e^{i(kx+ct)}, \end{array}$$ (32)

where *k* is the wave number of normalized form. Putting (32) in (31) and then solving for *c*, we obtain

$$\begin{array}{c} c=-\frac{\alpha k+\beta H_{0}^{2}k}{1+\gamma k^{2}}. \end{array}$$ (33)

It is evident from Eq (33) that any superposition of the solutions will exhibit a decaying behavior for negative values of *c*, indicating stable dispersion. The dispersion would be unstable otherwise.

## 6. Parametric effects on the wave profiles

**Fig 7:**
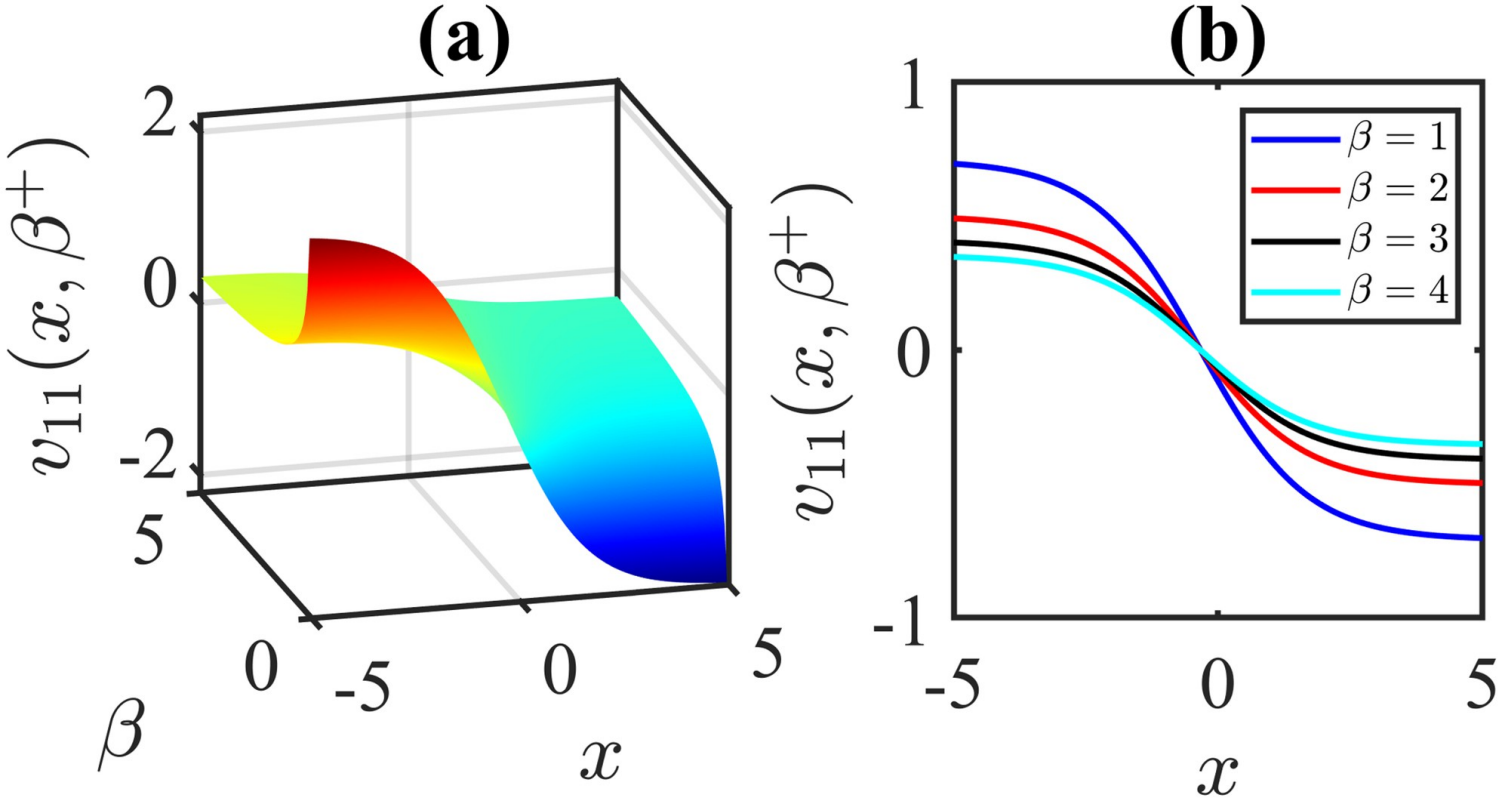
Anti-kink wave profile of (20): Effects of the parameter *β* on the wave profile for the values of *α* = −0.5, *l* = 1, *ω* = 5, *γ* = 1, *μ* = 2, *ν* = 3, *A* = 0, *B* = 1 at time *t* = 1.

**Fig 8:**
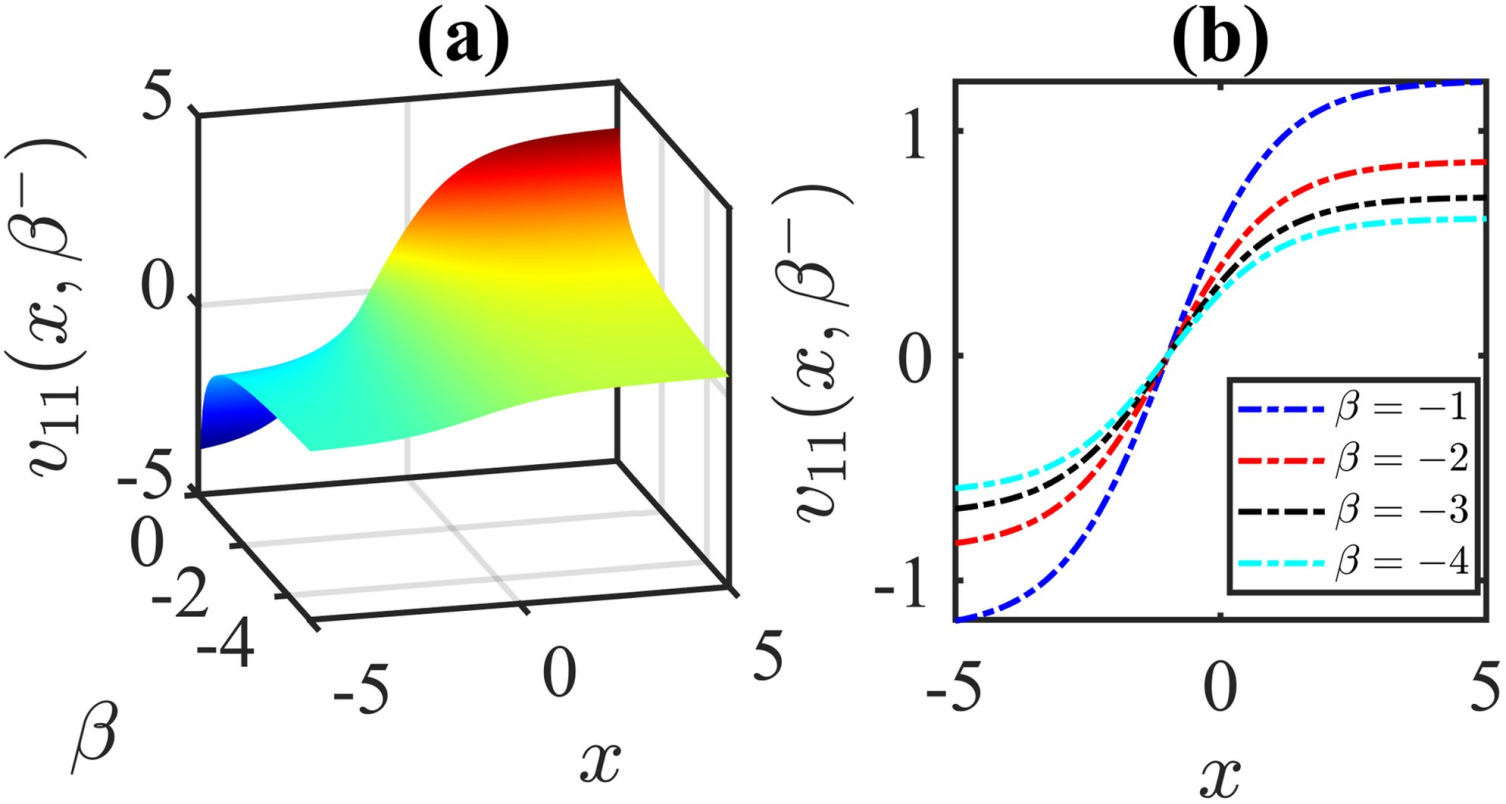
Kink wave profile of (20): Effects of the parameter *β* on the wave profile (20) for the values of *α* = −0.5, *l* = 1, *ω* = 5, *γ* = −1, *μ* = 2, *ν* = 3, *A* = 0, *B* = 1 at time *t* = 1.

**Fig 9:**
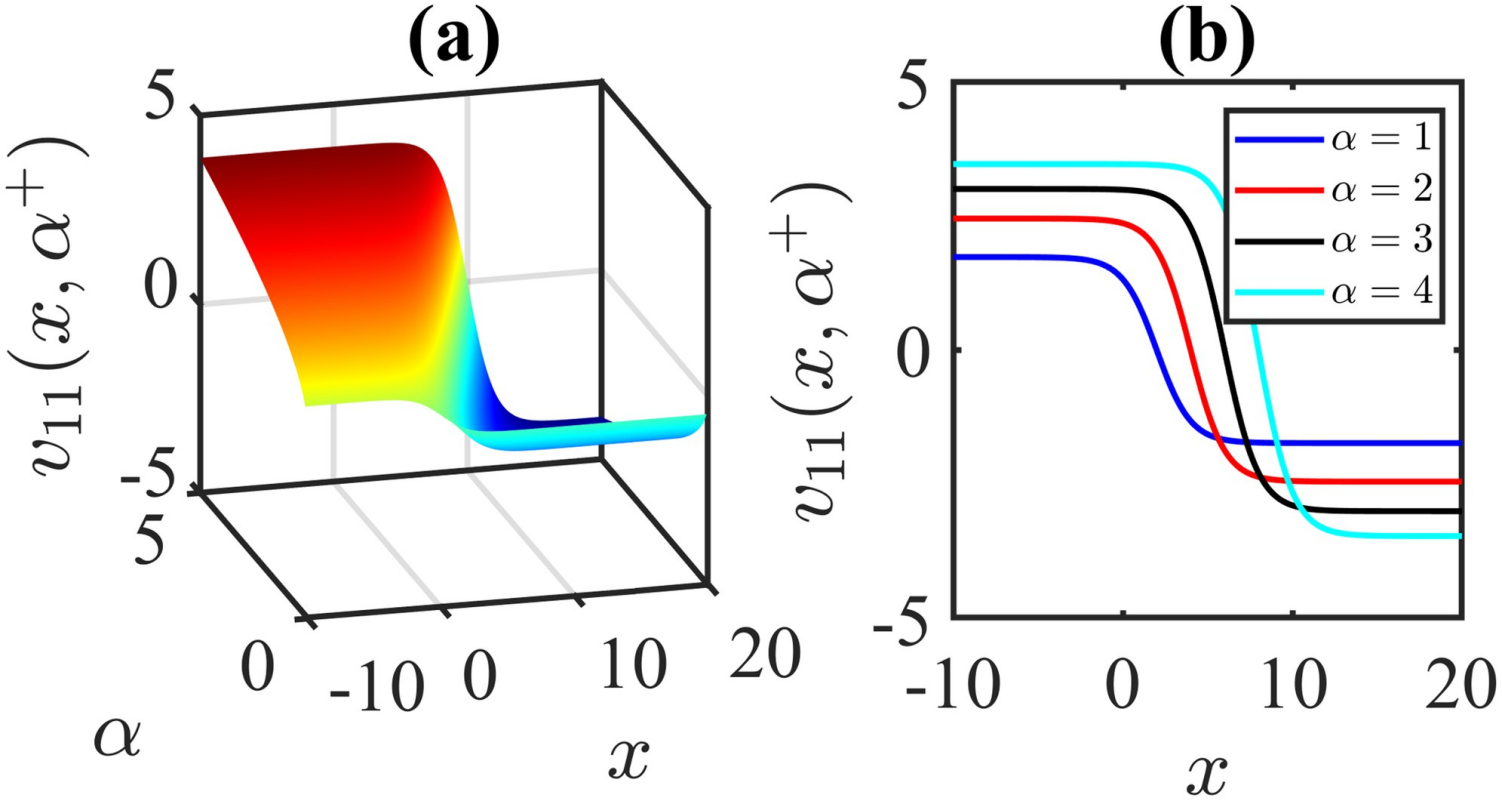
Anti-Kink wave profile of (20): Effects of the parameter *α* on the wave profile (20) for the values of *β* = 1, *l* = 1, *ω* = 5, *γ* = −1, *μ* = 2, *ν* = 3, *A* = 0, *B* = 1 at time *t* = 1.

**Fig 10:**
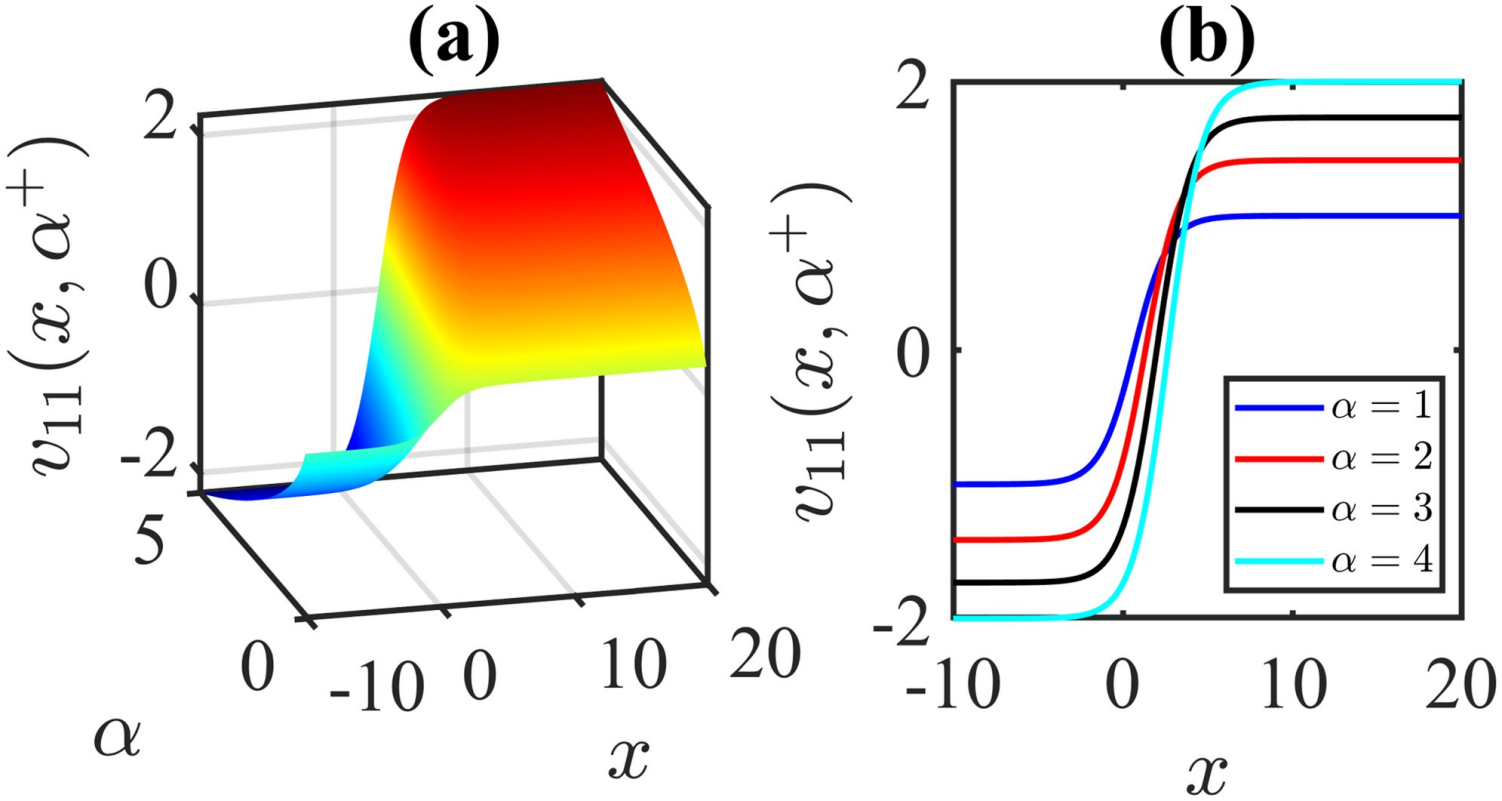
Kink wave profile of (20): Effects of the parameter *α* on the wave profile (20) for the values of *β* = −1, *l* = 1, *ω* = 5, *γ* = 1, *μ* = 2, *ν* = 3, *A* = 0, *B* = 1 at time *t* = 1.

**Fig 11:**
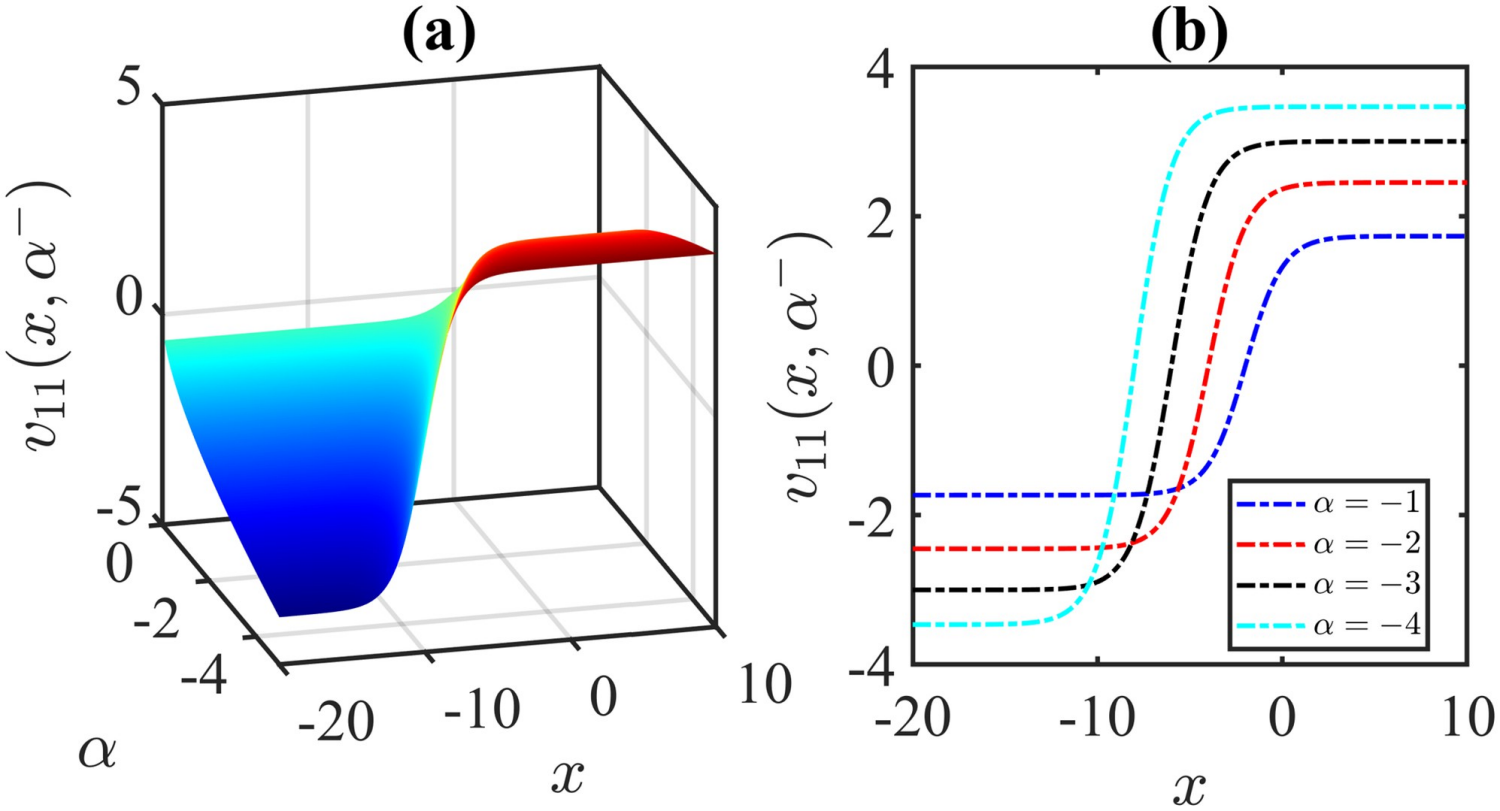
Kink wave profile of (20): Effects of the parameter *α* on the wave profile (20) for the values of *β* = −1, *l* = 1, *ω* = 5, *γ* = −1, *μ* = 2, *ν* = 3, *A* = 0, *B* = 1 at time *t* = 1.

**Fig 12:**
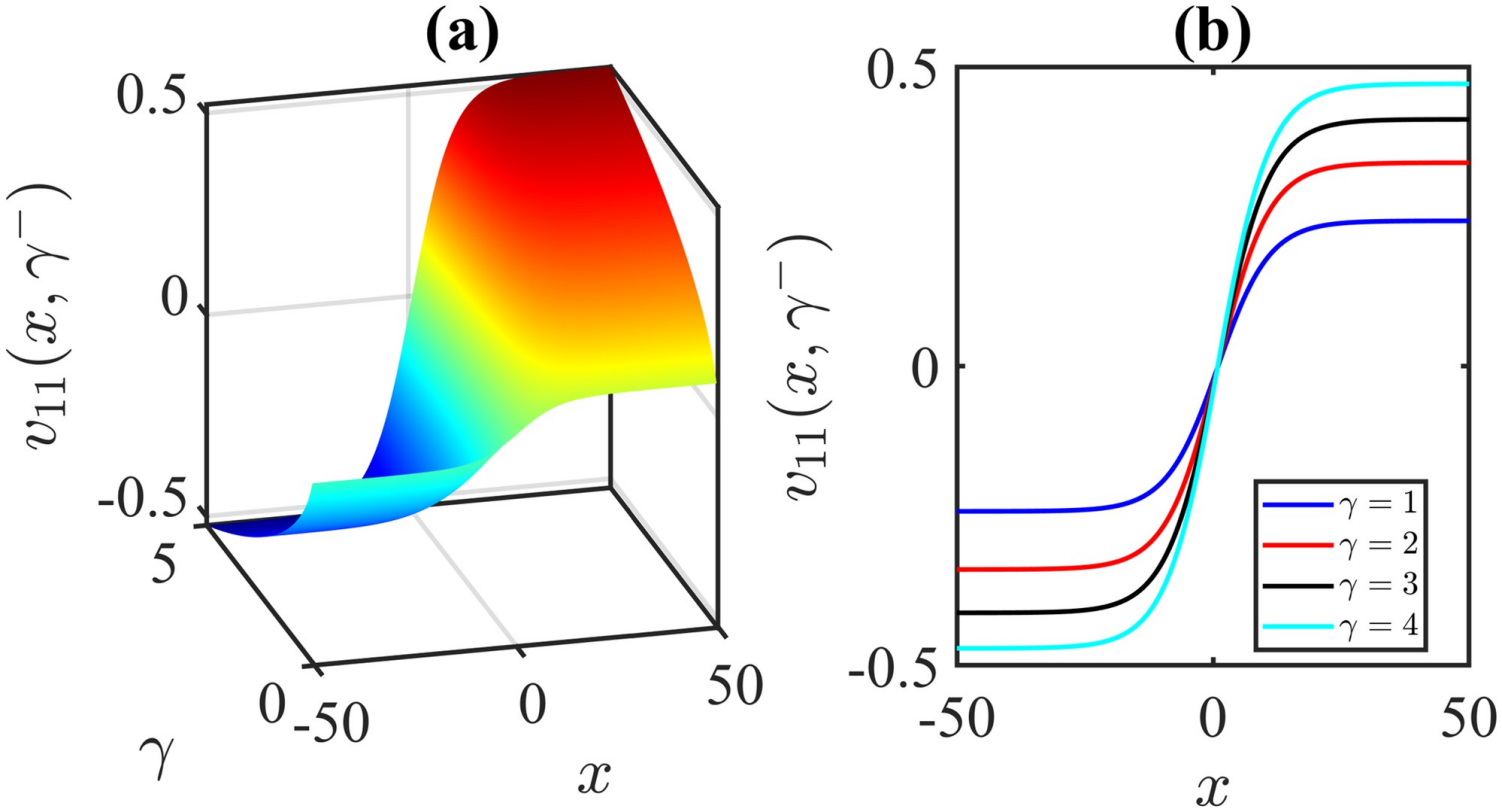
Kink wave profile of (20): Effects of the parameter *γ* on the wave profile (20) for the values of *α* = 1, *β* = −1, *l* = 0.2, *ω* = 5, *μ* = 2, *ν* = 3, *A* = 0, *B* = 1 at time *t* = 1.

**Fig 13:**
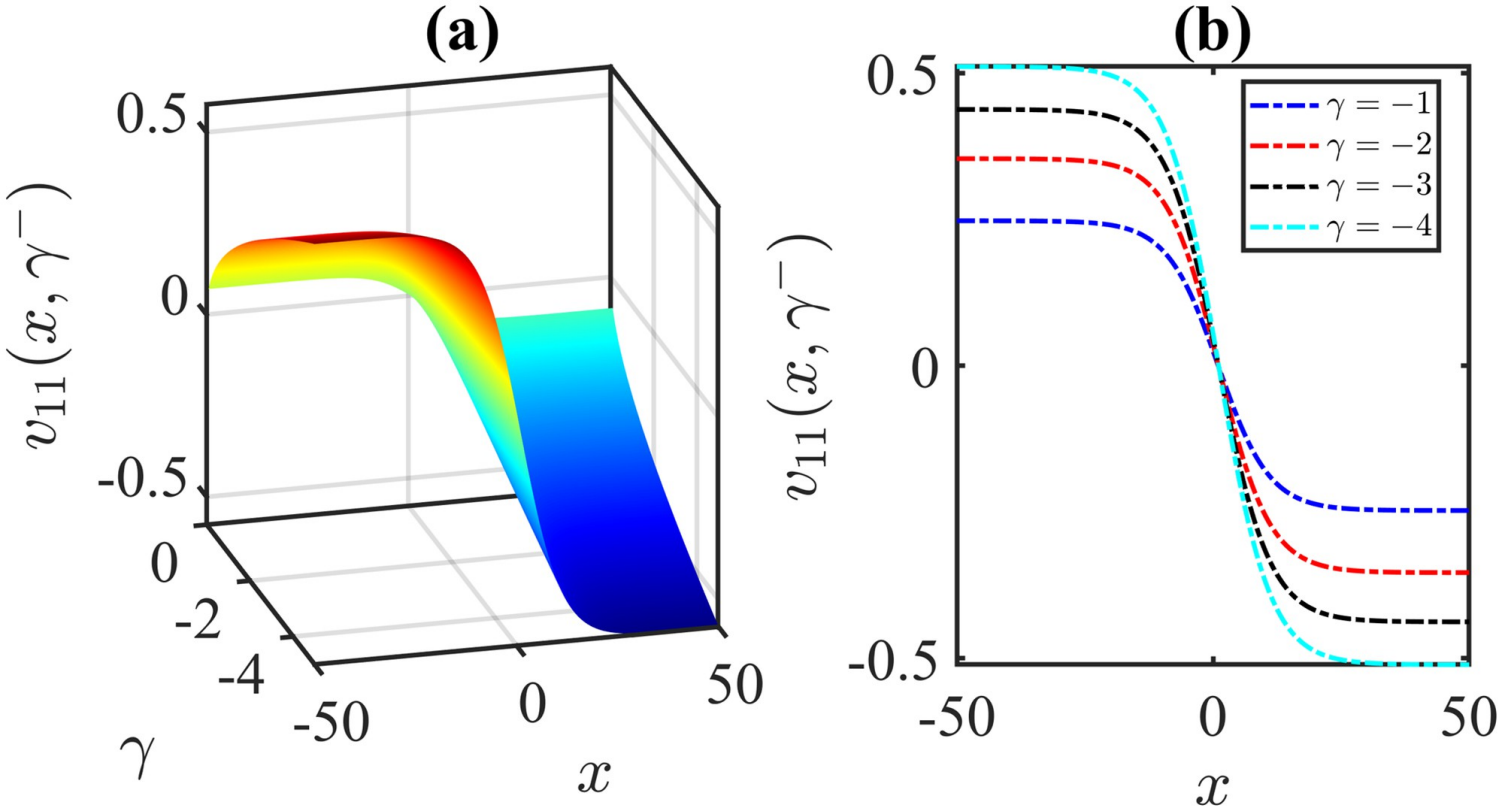
Anti-Kink wave profile of (20): Effects of the parameter *γ* on the wave profile (20) for the values of *α* = 1, *β* = 1, *l* = 0.2, *ω* = 5, *μ* = 2, *ν* = 3, *A* = 0, *B* = 1 at time *t* = 1.

**Fig 14:**
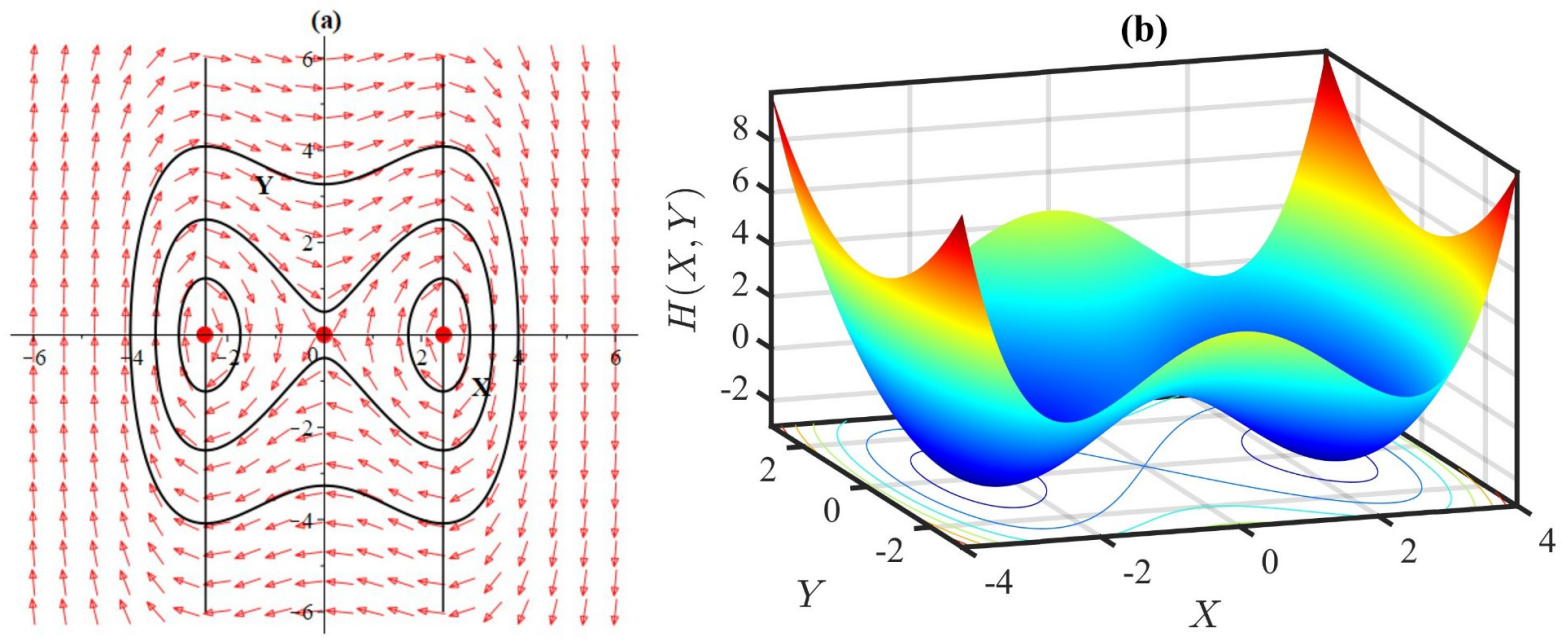
(a) Phase portrait with equilibria at $\left(0,0\right),\left(\pm \sqrt{6},0\right)$ of the system (34), and (b) 3D plot of the Hamiltonian (35), for *α* = 1, *β* = −1, *l* = 1, *ω* = 1, *γ* = 1.

**Fig 15:**
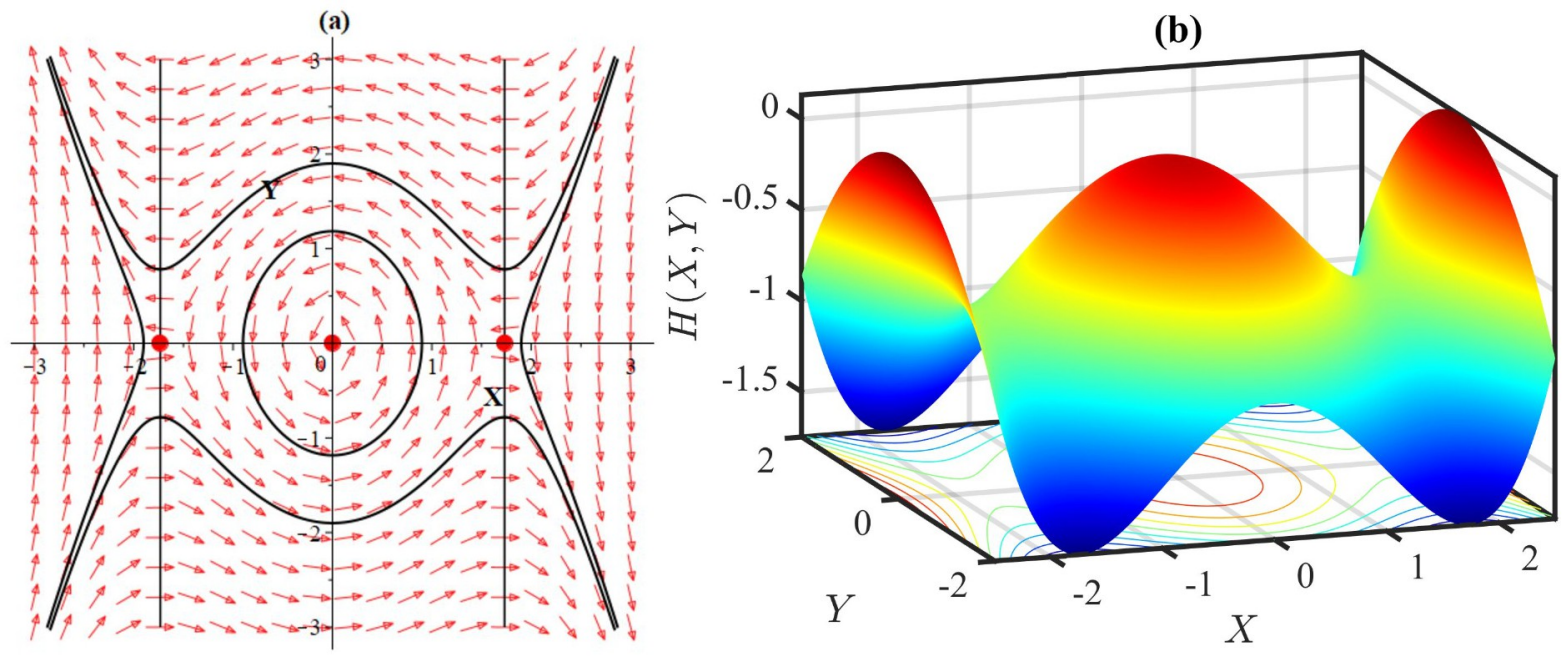
(a) Phase portrait with equilibria at $\left(0,0\right),\left(\pm \sqrt{3},0\right)$ of the system (34), and (b) 3D plot of the Hamiltonian (35), for *α* = −1, *β* = −1, *l* = 1, *ω* = 2, *γ* = −1.

**Fig 16:**
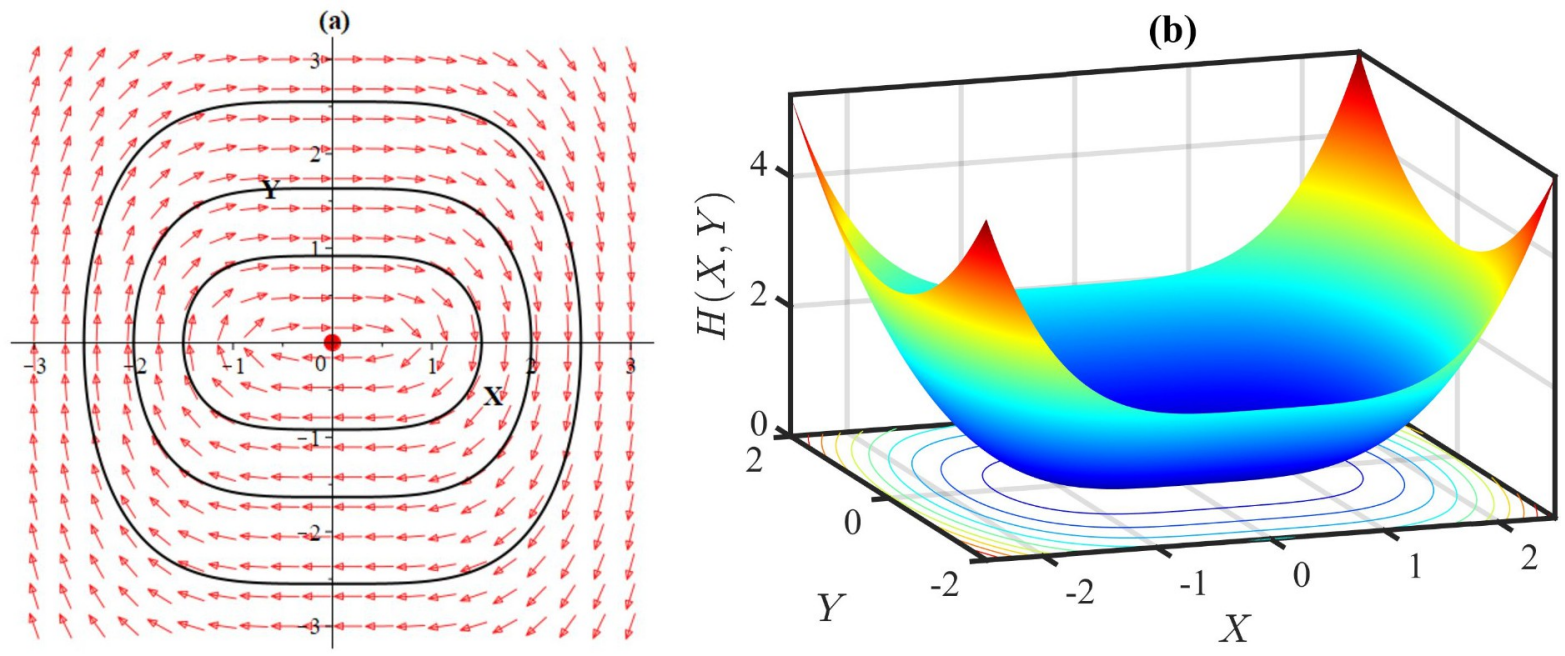
(a) Phase portrait with equilibrium at (0, 0) of the system (34), and (b) 3D plot of the Hamiltonian (35), for *α* = −1, *β* = −1, *l* = 1, *ω* = 1, *γ* = 1.

**Fig 17:**
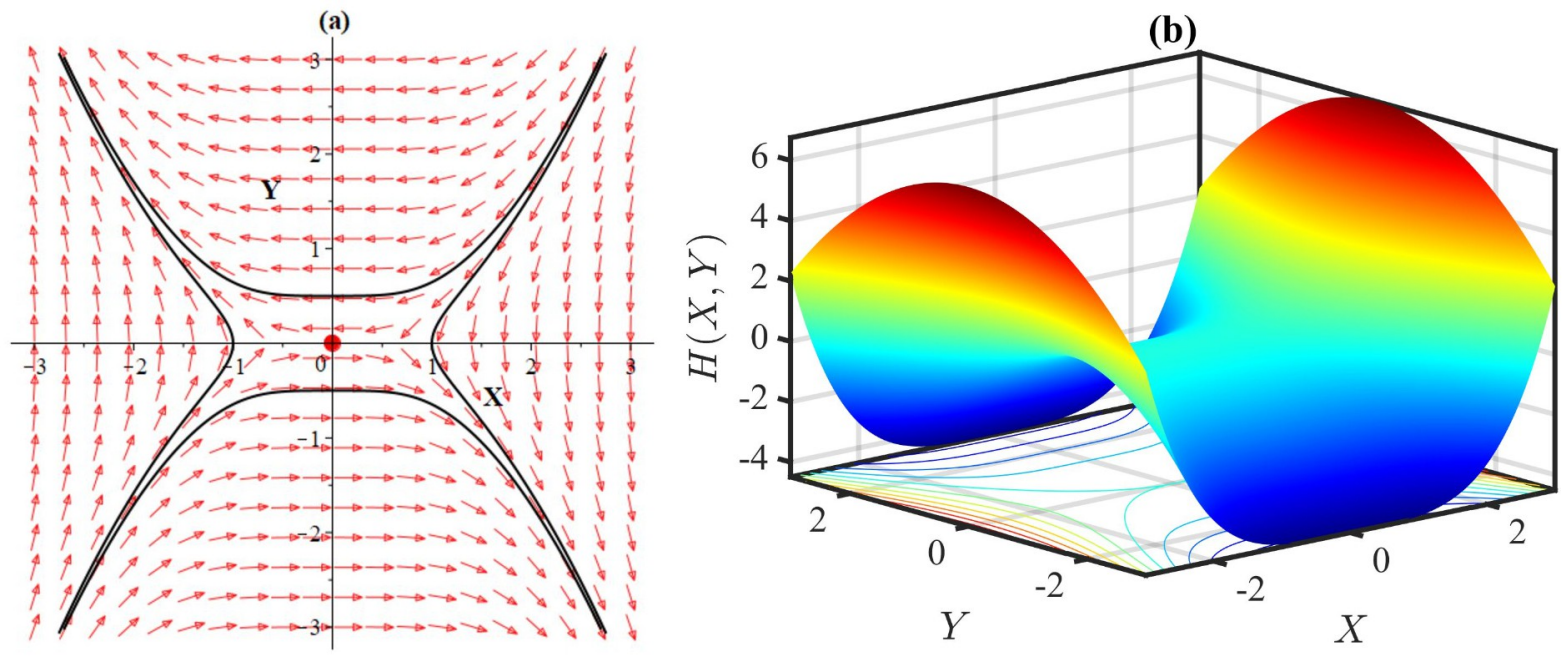
(a) Phase portrait with equilibrium at (0, 0) of the system (34), and (b) 3D plot of the Hamiltonian (35), for *α* = −1, *β* = −1, *l* = 1, *ω* = 1 *γ* = −1.

The importance of the model parameters in the mBBM equation is pivotal in determining the behaviour and temporal evolution of solutions. By delving into the examination of the influence of parameters, we aim to offer valuable insights into the dynamics of waves. The model parameters, *α*, *β*, *γ*, substantially impact various aspects of the system, including stability, bifurcation phenomena (Figs 14–17), wave propagation, amplitude modulation (Figs 7–13), and the overall dynamics. A profound comprehension of the effect of the parameters are imperative for both theoretical analysis and practical applications. To visually depict the impact of the parameters, Figs 7–13 present illustrations of amplitude modulation on the solution curves, namely *v*_11_(*x*, *t*). These figures serve as visual aids in understanding how parameters shapes the behavior of the solutions in the given context. The divergence of the nonlinear coefficient *β* towards either positive or negative infinity indicates a corresponding decrease in wave amplitude *v* to zero. Conversely, as the linear coefficients (*α*, *γ*) approach positive or negative infinity, they lead to an augmentation in wave amplitude. Specifically, a positive value for *β* results in the formation of anti-kink waves, while a negative value for *β* yields in the generation of kink waves. Analogously, the influence of model parameters on alternative solutions can be demonstrated in a similar manner.

## 7. Phase plane analysis

Varying parameters can cause changes in the qualitative structure of the flow. This can result in the creation or destruction of fixed points, or changes in their stability. Examining the movement of orbits in a phase plane can be highly informative in understanding the dynamics of the system and can assist in predicting its future behaviour. By analysing both the behaviour of the orbits in the phase plane and the corresponding solutions of the components concerning the wave variable, one can gain a comprehensive understanding of the dynamics of the system and make predictions about its future behaviour. We initiate the phase plane analysis [49] of the mBBM model, following the assumption that *W* = *X* and $X^{\prime }=\frac{Y}{\omega l^{2}\gamma }$. Then we can express Eq (9) in the form of a first-order dynamical system as follows:

$$\{\begin{array}{l} \frac{dX}{d\zeta }=\frac{Y}{\omega l^{2}\gamma }=g(X,Y),\\ \frac{dY}{d\zeta }=(\omega +l\alpha )X+\frac{l\beta }{3}X^{3}=h(X,Y). \end{array}$$ (34)

The Hamiltonian associated with the planar system (34) is

$$\begin{array}{c} H\left(X,Y\right)=-(\frac{\omega +l\alpha }{2})X^{2}-\frac{l\beta }{12}X^{4}+\frac{1}{2\omega l^{2}\gamma }Y^{2}. \end{array}$$ (35)

If *ω* = −*lα*, the system (34) possesses a single equilibrium point at (X*, Y*) = (0, 0). However, if *ω* ≠ −*lα*, the system exhibits three equilibrium points: (0, 0), $\left(\sqrt{-\frac{3(\omega +l\alpha )}{l\beta }},0\right)$ and $\left(-\sqrt{-\frac{3(\omega +l\alpha )}{l\beta }},0\right)$. It is to be noted that *lβ* ≠ 0.

The Jacobian matrix is defined as

$$J\left(X,Y\right)=[\begin{array}{cc} g_{X} & g_{Y}\\ h_{X} & h_{Y} \end{array}]=[\begin{array}{cc} 0 & \frac{1}{\omega l^{2}\gamma }\\ \omega +l\alpha +l\beta X^{2} & 0 \end{array}].$$

The corresponding charactersitic equation of the matrix *J* is

$$\lambda^{2}-\text{trace}\left(J\right)\lambda +\text{det}\left(J\right)=0,$$

where trace(*J*) = 0 and $\text{det}\left(J\right)=-\frac{\omega +l\alpha }{\omega l^{2}\gamma }-\frac{l\beta }{\omega l^{2}\gamma }X^{2}$. To examine the stability [49, 50] of the equilibrium points, we consider the following notations:

$$p=\text{trace}\left(J\right),\;q=\text{det}\left(J\right)\;\text{and}\;\Delta =p^{2}-4q.$$

**Case 1:** Stability of (0, 0)

For this case we have *p* = 0, $q=-\frac{\omega +l\alpha }{\omega l^{2}\gamma }$ and $\Delta =\frac{4(\omega +l\alpha )}{\omega l^{2}\gamma }$. If $\frac{\omega +l\alpha }{\omega l^{2}\gamma }>0$, i.e., *q* < 0, then the fixed point (0, 0) is a saddle point that is unstable in nature. If $\frac{\omega +l\alpha }{\omega l^{2}\gamma }<0$, i.e., Δ < 0 and *q* > 0, then the equilibrium is a stable center. Hence, the variations in the parameter values have the potential to shift the stability of the equilibrium point (0, 0), transforming it from an unstable saddle to a stable center (Figs 14–17).

**Case 2:** Stability of $\left(\pm \sqrt{-\frac{3(\omega +l\alpha )}{l\beta }},0\right)$

In this case we have *p* = 0, $q=\frac{2(\omega +l\alpha )}{\omega l^{2}\gamma }$ and $\Delta =-\frac{8(\omega +l\alpha )}{\omega l^{2}\gamma }$. If $\frac{\omega +l\alpha }{\omega l^{2}\gamma }<0$, then we have *q* < 0, indicating that the stationary points $\left(\pm \sqrt{-\frac{3(\omega +l\alpha )}{l\beta }},0\right)$ are unstable saddles. If $\frac{\omega +l\alpha }{\omega l^{2}\gamma }>0$ then we have Δ < 0 and *q* > 0 which means that the equilibria are stable centers. Therefore, the change in the parameter values have the potential to shift the stability of the equilibrium points $\left(\pm \sqrt{-\frac{3(\omega +l\alpha )}{l\beta }},0\right)$, transforming them from unstable saddle to stable center (Figs 14–15).

## 8. Conclusion

In this study, we delved into the investigation of the mBBM equation utilizing the rational $\left(\frac{G^{\prime }}{G}\right)$-expansion method. Our examination led to the discovery of a rich array of soliton solutions, including kink, anti-kink, anti-peakon, singular kink, singular periodic, singular bell, dark, and V-shaped solitons. These diverse solutions serve as windows into the intricate physical behaviors inherent in the governing model, offering valuable insights for understanding its dynamics. To ensure the credibility of our findings, we rigorously cross-verified the obtained solutions by substituting them into the original differential equation, leveraging mathematical software, such as Maple. This meticulous validation process underscores the robustness of our results. Moreover, we systematically explored the influence of various parameters on the wave profile of the governing equation through the generation of 2D and 3D plots (please see Figs 7–13). Notably, our simulations revealed intriguing patterns of the wave profile in the behavior of the nonlinear wave coefficient *β* and the linear coefficients (*α*, *γ*) as they approach positive or negative infinity. Specifically, we observed a consequential reduction in wave amplitude *v* to zero with increasing asymptotic values of *β*, while the linear coefficients exhibited an amplification in wave amplitude under similar conditions (please see Figs 7–13). These insights deepen our understanding of the underlying mechanisms governing the mBBM model and its response to parameter variations.

A rigorous validation process using mathematical software Maple, coupled with the comprehensive analysis of parameter influences on the wave profile, enhances the credibility and novelty of our results. Additionally, our exploration of stability considerations and phase plane analysis adds another layer of original contribution to the study. By elucidating the influence of parameter variations on the dynamics of soliton solutions, we uncover novel insights into the behavior of the mBBM model, further distinguishing our work from existing research.

In essence, our study contributes to the comprehensive understanding of the mBBM equation and its soliton solutions, elucidating the intricate interplay between parameters and wave dynamics. These findings hold implications for diverse fields where nonlinear wave phenomena are encountered, offering avenues for further exploration and application.
